# PROTOCOL: Interventions Targeting Misinformation, Disinformation and Malinformation for Reducing and Countering Violent Extremism: A Systematic Review

**DOI:** 10.1177/18911803261459099

**Published:** 2026-06-21

**Authors:** Cátia Moreira de Carvalho, João Pedro Ramos, Catarina Simas, Pedro Barbosa, Sarah Carthy, Marta Pinto

**Affiliations:** 1 School of Law and Governance, 8818 Dublin City University, Dublin, Ireland; 2 Portuguese Institute of International Relations, NOVA University of Lisbon, Lisbon, Portugal; 3 Faculty of Psychology and Education Sciences, 166419 University of Porto, Porto, Portugal; 4 EPIUnit ITR, Instituto de Saúde Pública da Universidade do Porto, Porto, Portugal; 5 School of Medicine and Biomedical Sciences, 26706 University of Porto, Porto, Portugal; 6 Institute of Security and Global Affairs, 194981 Leiden University, Den Haag, Netherlands; 7 Health Research Network: From the Lab to the Community, 26706 University of Porto, Porto, Portugal

**Keywords:** Disinformation, Malinformation, Misinformation, Extremism, Violent extremism

## Abstract

The dissemination of false or inaccurate information, and its subsequent effect on behaviour, is not a new phenomenon. However, in recent years, alongside the emergence of multiple methods of information transmission, the speed, scale and volume of mis-, dis-, and malinformation (MDM) has reached a new threshold, contributing to societal polarisation and mistrust towards authorities. It has been postulated that the phenomenon may also play a role in the psychosocial process of *radicalisation*. By re-framing grievances or events and creating shared identities or networks amongst ‘believers’, exposure to MDM may interact with other audience vulnerabilities to facilitate radicalisation towards violent extremist narratives and networks. This systematic review will examine interventions designed to counter or reduce the effects of exposure to false information and their impact on violent extremism. The review will synthesise evidence across diverse methodologies and settings, identify gaps in the literature, and highlight best practices for reducing or countering the effects of false narratives and their subsequent influence on violent extremist behaviour and attitudes. The findings will inform policymakers, educators, and practitioners about actionable measures to combat the harmful consequences of false information in the context of violent extremism.

## Background

The dissemination of false or inaccurate information, and its subsequent effects on human behaviour, is not a new phenomenon. In medicine, information environments comprised of “facts mingle[d] with half-truths and untruths” (Rojecki & Meraz, 2016, p. 25) have always existed, playing a role in behavioural outcomes ranging from low vaccine uptake (Jolley & Douglas, 2014a) to the use of apple cider vinegar to treat rosacea and hypertension (Chen et al., 2021). In 2020, the World Health Organisation described the phenomenon as an “infodemic” (as cited in U.S. Surgeon General, 2021, p. 4), prompting a growing body of literature on how to reclaim health communication (Armstrong & Naylor, 2019; Walter et al., 2021). However, the dissemination of false or misleading information is not exclusive to healthcare, nor are its consequences limited to health behaviours.

The adoption of belief systems informed by false information is associated with a higher carbon footprint (Jolley & Douglas, 2014b), workplace disengagement (Douglas & Leite, 2017), and even stronger intentions to partake in crime (Jolley et al., 2019). The phenomenon has also been found to influence how individuals perceive outgroup members, including their levels of prejudice (Jolley et al., 2020; Kofta et al., 2020), voting behaviour (Cantarella et al., 2023), and support for restrictions on others’ civil liberties (Kim & Park, 2023; Price & Hsu, 1992). Exposure to false or misleading information has also been found to influence attitudes towards politicians (Ahmed & Gil-Lopez, 2024) and, depending on your political persuasion, trust in government (Ognyanova et al., 2020). In recent years, it has also been postulated to play a role in radicalisation to violent extremist belief systems (Schumann et al., 2024).

In 2016, a story circulated across social media that a pizzeria in Washington, DC, was imprisoning children as part of a child sex trafficking ring connected to Hilary Clinton. A 28-year-old man drove from North Carolina to the restaurant to investigate the claim and, after searching and finding nothing, opened fire. Although nobody was injured, the “Pizzagate” incident represented a novel strain of radicalisation emerging amongst disenfranchised, white men (Johnson, 2018), one which relied heavily on reframed or out-of-context interpretations of history (Strick, 2022) and false or misleading evidence (Juhász & Szicherle, 2017) to elicit strong emotions (Zollo et al., 2015), and, too often, prompting violence.

There are three conceptual distinctions which are central to this review: *misinformation*, *disinformation* and *malinformation*. These concepts are distinguished from each other, to varying degrees, by *truth* and *intentionality,* thus providing conceptual clarity. In terms of intentionality or the “agent” at play (Wardle & Derakhshan, 2017, p. 20), information which is false or misleading, but disseminated without the intention of manipulating or causing harm (e.g., through mistake, negligence, or bias, see Fallis, 2010, p. 1; Bodaghi et al., 2023), is defined as *misinformation* (Ireton & Posetti, 2018; Wardle & Derakhshan, 2017). When we observe how individuals have, throughout history, come to internalise and propagate ill-informed attitudes which they believe to be accurate (Dovido et al., 2010; Eagly & Diekman, 2005), it is clear that the theoretical fulcrum of misinformation is well established in socio-cognitive frameworks such as Social Dominance Theory (Sidanius & Pratto, 1999), Social Identity Theory (Tajfel & Turner, 1979), and Self-Categorisation Theory (Turner, 1985; see also Onorato & Turner, 2001).

*Disinformation* is defined as the deliberate use of erroneous information to manipulate, cause harm or confuse people (Bodaghi et al., 2023; Floridi, 2011), mirroring comparable phenomena defined by malicious intent, such as “propaganda” (Jowett & O’Donnell, 2012, p. 6). In other words, it is the intention of the communicator, rather than the processing of the information, which distinguishes it from misinformation. In this way, disinformation can also be understood through the socio-cognitive frameworks outlined above, as well as traditional “persuasion” (Miller, 1980, p. 1) models such as the Elaboration Likelihood Model (Petty & Cacioppo, 1986), and resistance theories (McGuire, 1961; Moyer-Gusé, 2008). *Malinformation*, however, refers to the dissemination of correct information out of context for deceptive ends. In other words, it is distinguished from the others by the veracity of the information itself. It involves a “repurposing of the truth” (Baines & Elliott, 2020, p. 12), which, like disinformation, closely maps onto traditional understandings of persuasion and propaganda but is technically true in nature.

In their pedagogical response to misinformation as a tool to “catalyse ideologically motivated violence”, Joshi and Sabic-El-Rayess (2023, p. 97) illustrate how this type of misinformation represents a vastly different challenge than a decade ago. Through the emergence of multiple methods of information transmission, not only can false or misleading information reach audiences at greater speed and quantity (Blumler, 2015; Kapantai et al., 2020; Niklewicz, 2017), but it often spreads “farther, faster, deeper and more broadly” than the truth (Vosoughi et al., 2018, p. 1146). This is attributed to the “hedonic” nature of social media as well as the overall communication architecture (Muhammed & Mathew, 2022, p. 272). In other words, not only are audiences less likely to verify the information they come across (Oh et al., 2013), but the rise of “multi-sensory” (e.g., images, podcasts, short videos) methods of transmission (Ekström & Westlund, 2019, p. 100) further facilitates the sharing of false or misleading information. Platforms such as WhatsApp have even had to limit forwarding functions to counteract this phenomenon (Hern, 2020).

### Mis-, Dis-, and Malinformation (MDM) and Violent Extremism

Radicalisation is a process characterised by increasing adherence to “extremist” beliefs (Borum, 2011) or, in other words, a set of beliefs which legitimise hostile action against an adversarial group (Berger, 2018). This increasing adherence may include the justification of political violence or terrorism (Haleem & Masood, 2023; Ozer & Bertelsen, 2018), but radicalisation, by definition, does not always prompt congruent behaviour (McCauley & Moskalenko, 2017), violence or otherwise (Schuurman & Carthy, 2023). The process by which an individual becomes radicalised is theorised to implicate a range of structural, group and individual-level factors (Jensen et al., 2018). However, across a multitude of disciplines, a central tenet of radicalisation scholarship is its *communication* underpinnings.

Not only is the manipulation of complex realities into simplistic stories a key feature of violent extremist recruitment (e.g., Halverson et al., 2011; Marcks & Pawelz, 2022; McAuley & Ferguson, 2016), but when these “narratives” combine with other influences (Kundnani, 2012, p. 18), both at the individual level and as a result of broader, socialisation processes, it is well established that these pillars combine to facilitate the radicalisation process (Kruglanski et al., 2014). However, while the reduction of complex realities into simplistic narratives can be understood as a radicalisation mechanism or pillar, the role played by false or misleading information *within* this pillar is a complex one.

First, it is important to emphasise that narratives which promote radicalisation are, by no means, composed entirely of false or misleading information. As Schmid (2014) reminds us, the strength of Al Qaeda’s narrative lay in its incorporation of genuine grievances held by Muslims worldwide. However, even if the information is “not false by definition”, it may be “unverified, implausible and epistemically unsound” (e.g., conspiracy theories, see Brotherton & French, 2015, p. 1). and, depending on the intent of the communicator, could be classified as either misinformation, disinformation or malinformation. However, while elements of truth are often woven into extremist narratives, many purposely incorporate barely believable elements to better elucidate two key elements: “a grievance and a culprit” (Kruglanski et al., 2022, p. 3). In this way, false or misleading information plays a role in crafting a particular social construction, one which often interferes with previously held beliefs, making recipients “less confident” about their pre-exposure narrative (Dalton & Daneman, 2006, p. 487). The manipulated narrative may also interact with various audience vulnerabilities such as perceived grievances, low social integration, self-uncertainty (Roberts-Ingleson & McCann, 2023), lack of knowledge or understanding on a particular topic (Kata, 2010 as cited in Featherstone & Zhang, 2020), or even weaning trust in authorities or institutions (Jolley & Douglas, 2017). While the interaction between narrative and vulnerability has been more substantively studied in the context of healthcare (e.g., vaccine misinformation, see Featherstone & Zhang, 2020), there are comparable findings in the radicalisation literature, particularly in observing the ease with which vulnerable people can be targeted via digital networks (Ekström & Westlund, 2019).

In the communication literature, one’s disposition towards trait anxiety affects how information is perceived, and this is central to understanding why false or misleading information may resonate with certain audiences. Those high in trait anxiety are more likely, for instance, to adhere to conspiratorial narratives about Jewish people (Grzesiak-Feldman, 2013), and this vulnerability is also well established in the radicalisation literature. When exposed to vignettes depicting “terroristic content”, McGarry and Shortland (2023, p. 1) found that the motivation to engage further with the material was particularly salient for those who were predisposed towards feeling anxious. Kruglanski et al. (2022) propose that simplistic stories about protagonists and antagonists will satisfy a basic need in the target, offering a remedy to feelings of existential anxiety, and triggering radicalisation through a “quest for significance” (p. 3). It may be the case that the tendency to believe in “controversial” stories that contain false or misleading information is rooted in a broader existential threat (Newheiser et al., 2011, p. 1007), one which connects to one’s desire for control and security (Douglas et al., 2017). This may become salient when information relating to antagonists is portrayed in threatening ways.

However, radicalisation is not exclusively concerned with the relationship between stories and individual vulnerabilities. Since its inception, radicalisation scholars have proposed that broader, extremist networks play an integral role in various radicalisation pathways (Della Porta, 2013; Sageman, 2011). Whether the connection is real (Silke, 2003) or digital (Conway, 2016), a network of like-minded individuals can offer opportunities to satisfy needs, as well as provide rewards (Webber & Kruglanski, 2018) and the creation of such networks has also been observed in how false or misleading information resonates with audiences. Ultimately, misinformation creates opportunities for “political polarisation” (Piazza, 2022, p. 57); those who accept the information as true represent a cohesive network of like-minded individuals, whereas disbelievers represent a “judgement[al], disparag[ing]” out-group (Booth et al., 2024, p. 447). For example, the widespread proliferation of QAnon’s cabal story, which was mentioned in the previous section (see also Harari, 2020; Johnson, 2018), suggests that QAnon helped craft a network of “white masculinist paranoia”, one which “radicalise[d] white men” (p. 102).

While the radicalisation mechanisms activated by false or misleading information are theoretically important, it is also important to draw attention to the action tendencies associated with exposure to this type of information and elucidate why it is of concern to those tasked with countering or preventing violent extremism. Exposure to MDM ultimately creates lasting misperceptions, and these can prompt feelings of injustice through, for instance, anger (Carver & Harmon-Jones, 2009; Featherstone & Zhang, 2020). Those who adhere to the belief that 5G mobile technology is associated with COVID-19, for example, have been found to endorse violence (Jolley & Paterson, 2020) and, while behaviour emerging from such trajectories is not always violent in nature (Kruglanski et al., 2022; Schuurman & Carthy, 2023), some adherents have engaged in destructive actions such as vandalism and arson (Lewandowsky & Cook, 2020). A small number have also been involved in fatal attacks against authorities (Gillespie & McGowan, 2022; as cited in Booth et al., 2024). In this way, MDM represent malleable tools at the disposal of violent extremist networks, ones which can manipulate existing anxieties, and prompt a range of behavioural responses, including manifestations of extreme violence. For this reason, understanding and dismantling this new “information landscape” (Amarasingam & Argentino, 2020, p. 42) has become particularly relevant to those tasked with preventing or countering this type of audience manipulation.

MDM can amplify violent behaviour by distorting perceptions, heightening emotional responses, and fostering social polarisation. False or misleading narratives can dehumanise targeted groups, exaggerate threats, and attribute blame inaccurately, thereby inciting anger, fear, and hostility that lower thresholds for violent actions (Braddock & Horgan, 2015). These dynamics contribute to deepened societal divisions, reinforcing echo chambers and “us versus them” mentalities that legitimise aggression and violence (Sunstein, 2009). Moreover, MDM undermines trust in public institutions and authorities, potentially driving disenfranchised individuals toward radicalisation and violent extremism as alternative means of addressing grievances (Neumann, 2013). Extremist groups can exploit MDM to mobilise supporters, justify violent acts, and coordinate activities, often using online platforms to amplify their reach and impact (Conway, 2016). Therefore, addressing MDM is critical for preventing the escalation of violent behaviour and safeguarding social cohesion.

### Actions to Prevent and/or Counter MDM

This section primarily focuses on governmental interventions aimed at addressing MDM, reflecting the significant role that governments and political institutions play in shaping policies and frameworks to mitigate the risks posed by MDM. This focus is warranted due to the unique authority and responsibility governments have in safeguarding public trust, democratic stability, public health, and human rights, areas severely threatened by the spread of MDM (Ireton & Posetti, 2018; Watts et al., 2021). To tackle MDM, governments and political institutions have been delivering policy and technical recommendations. The United States Government has taken a proactive step in enhancing its cybersecurity measures through the creation of the Cybersecurity and Infrastructure Security Agency (CISA) to build resilience to MDM, by promoting an understanding of the scale of the problem and fostering responses that may mitigate the risks and their impact (CISA, n/d). This strategy adopts a whole-of-society approach involving multiple stakeholders, by engaging MDM experts to contribute to tackling, preventing, mitigating and responding, as well as trusted voices, such as government officials, community leaders and civil society organisations, to better reach out to vulnerable communities. Furthermore, it seeks to empower the community as a whole to prevent and stop the dissemination of MDM by developing information tools for their own use (CISA, n/d).

A strengthened Code of Practice on Disinformation was developed in 2021 by the European Commission, which calls for a larger participation of relevant stakeholders. These stakeholders are sought as a means of contributing resources or expertise to the Code’s implementation, reducing funding for disinformation and promoting transparency and accountability. Stakeholders are expected to provide a comprehensive understanding of how MDM is disseminated, as well as train and capacitate users to identify MDM, including cooperation with fact-checkers and a strengthened monitoring framework, based on indicators measuring the outcomes and impact of MDM. All possible signatories are encouraged to participate and to be transparent on how they enforce these measures.

It is also important to mention that the United Nations Human Rights Council has adopted resolution 76/227 on “Countering disinformation for the promotion and protection of human rights and fundamental freedoms” to implement a plan of action that tackles disinformation. This resolution includes strengthening the relevance of free speech and its importance to counter MDM, and enhances the role of governments in promoting reliable information and transparency regarding official data (United Nations [UN], 2022).

At the same time, one should acknowledge the critical role of private sector actors, particularly large technology companies (commonly referred to as Big Tech), in content moderation on social media platforms. Although content moderation is not a governmental intervention per se, it directly influences the information ecosystem where MDM circulates. Given the pervasive influence of these platforms in shaping public discourse (Swastiningsih et al., 2024) their moderation policies and practices significantly impact the spread or containment of MDM. The ongoing global debate around regulating these practices underscores the need to consider them alongside governmental efforts, as effective responses require a multi-sectoral approach involving both public authorities and private entities (Morar & Santos, 2020).

By explicitly addressing both governmental and non-governmental actors, this section provides a brief overview of the institutional landscape confronting MDM. It demonstrates that governments and international organisations have an intent to produce actions and legislation to tackle MDM, and the inclusion of Big Tech’s role in content moderation reflects the interconnectedness of these efforts within the broader information ecosystem. Together, these actors contribute to building resilience against MDM by promoting reliable information, transparency, accountability, and by empowering communities to recognise and counter misinformation, disinformation, and malinformation (UN, 2022; Government of Canada, nd). This multifaceted approach is essential for safeguarding democratic processes, human rights, and social cohesion in contemporary societies (Harley & Cooper, 2021). The critical role that trustworthy and accurate information plays in shaping public opinion, informing civic engagement, and safeguarding democratic norms has underscored the importance of conducting a systematic review to assess the effectiveness of interventions designed to counter MDM in the context of preventing and reducing violent extremism (Harley & Cooper, 2021).

### The Intervention

This review will focus on interventions that target MDM to counter and reduce violent extremism. Although interventions in this field are a relatively recent phenomenon, there are several existing studies that illustrate the various types that these interventions can take (e.g., Braddock, 2019; Carthy & Sarma, 2023).

Reactive interventions, such as fact-checking and educational or informational approaches, primarily aim to mitigate the effects of MDM after it has spread. Their strength lies in providing corrective information and reducing misinformation’s immediate impact; however, these approaches often face challenges related to timeliness, scalability, and the potential for limited reach once false information has already permeated social networks (Arribas et al., 2025; Dickinson et al., 2025; Matanji et al., 2024).

Proactive interventions seek to prevent or reduce the dissemination and impact of MDM before it becomes widespread. These include pre-bunking and debunking, inoculation, fact-checking, media literacy programs, gamification, early detection systems, and educational efforts. Their purpose is to build cognitive resistance and enhance individuals’ ability to identify and critically evaluate MDM in advance. While proactive strategies can be more effective in reducing the overall spread of harmful content, they require sustained engagement and resources, and their impact can be difficult to measure in the short term (Harjani et al., 2022; Huang et al., 2024).

Overall, these interventions aim to strengthen the capacity of individuals to detect MDM, to build cognitive resistance, to form correct evaluation and identification of MDM, and ultimately to promote the ability to stop the dissemination of MDM by voluntary and involuntary users. These interventions can either be implemented online or offline (for example, in schools or universities), and tackle different groups of the population and age groups. For those who are not radicalised, these interventions can act as a preventative measure; for those who are vulnerable populations (i.e., who are already radicalised or at risk of becoming), these interventions aim to counter or reduce violent extremism.

The public health model against violent extremism is useful to understand these different stages. This model posits that preventing and countering violent extremism is divided into three stages (Bhui et al., 2012; Weine et al., 2016). The first is primary prevention, which focuses on community-wide strategies to build resilience to violent extremism, one of which is the support for building narratives that counter extremist ideologies. Secondary prevention aims to prevent vulnerable individuals from engaging in violent extremism. The tertiary prevention stage aims at developing strategies to disengage or deradicalise those individuals who are already engaged in violent extremism and to prevent them from committing violence. It may also include rehabilitation and reintegration strategies. While most MDM interventions fit within the primary prevention stage, some interventions can also be used with individuals at risk of radicalisation into violent extremism, (i.e., secondary prevention).

### How the Intervention Might Work

Interventions in this field are relatively recent, though some of its theoretical underpinnings have been used for a longer time. For instance, inoculation theory (McGuire, 1961) has been informing many different interventions. This theory posits that preemptive exposure to a mild version of a persuasive argument could impart psychological resistance against future persuasive attacks (McGuire, 1961). Indeed, inoculation is focused on preventing people from being influenced by misinformation before it spreads; that is, individuals are exposed to a mild version of a false claim to strengthen their defences against future misinformation. This theoretical framework is divided into three categories: Classic Inoculation, Warning, and Strategic Inoculation (which includes the Gamified Inoculation) (Ziemer & Rothmund, 2024). Classic Inoculation associates a mild false claim with a refutational preemption, giving the necessary means to allow the person to counterargue the falsehood (McGuire, 1961). More recently, some inoculation interventions combine the technique with media literacy [as a part of Boosting Literacy interventions, it aims to motivate reasoning within the digital context (Tseng et al., 2021; Ziemer & Rothmund, 2024)] and knowledge enhancement [an intervention that combats misinformation through the teaching of facts about a given topic (Roozenbeek et al., 2020; Ziemer & Rothmund, 2024;).

Strategy-based inoculations operate on the premise that inoculation can be effective even when individuals already possess existing misperceptions, and they might appear in combination with other strategies that also contribute to hindering the dissemination of MDM. The inoculation process involves two essential mechanisms: *threat* (or forewarning) and *refutational preemption* (prebunking). The threat component alerts individuals that they are likely to encounter a manipulative message, thereby activating their cognitive “immune system.” The second mechanism, refutational preemption or prebunking, equips individuals with counterarguments to effectively challenge and reject misleading claims. Together, these elements prepare individuals to resist more persuasive and stronger misleading information in the future (Traberg et al., 2022).

Nonetheless, it is to be noted that people become more resistant to deception once they realise that there is a vulnerability in them to be deceived, as well as being conscious of the intention of deception (Van der Linden et al., 2017). This kind of intervention reduces the extremist groups’ credibility perceived by the participant, as well as the intention to support them (Braddock, 2019). Inoculation, through the introduction of threats to the participants’ autonomy and values, allows an activation of mechanisms in order to protect their agency, according to the reactance theory (Brehm, 1966). Braddock (2019) posits that this reactance, as well as the lack of credibility attributed to the extremist propaganda, is the key to the effectiveness of such interventions. Short follow-up interventions, or feedback, can help maintain its effectiveness over time (Leder et al., 2024).

Another type of common intervention are digital literacy enhancement programmes, which often are also called media literacy. These refer to the ability to analyse and evaluate online information, including the reliability of sources and evidence (Ziemer & Rothmund, 2024). These programs are commonly integrated into formal education or community outreach, particularly for young or older people. These types of interventions are designed to improve people’s ability to evaluate the accuracy of information, to navigate online tasks and to differentiate fact and fiction online (Moore & Hancock, 2022).

Media literacy training can enhance the ability to recognise false information, though effectiveness varies across pedagogical approaches (Bateman & Jackson, 2024). The most successful models combine skills development with fostering confidence, responsibility, and proactive information seeking (Bateman & Jackson, 2024). However, scalability, timeliness, and targeting remain significant challenges, as reaching large and vulnerable populations is resource-intensive (Bateman & Jackson, 2024). Social media platforms are increasingly employing content labels, some of which are intended to assist users in evaluating the trustworthiness of information (Bateman & Jackson, 2024). Labelling refers to the provision of contextual information or advisories aimed at informing or shaping user interpretation of content, without engaging in direct fact-checking.

Other types of interventions have also been used, such as fact-checking through debunking (Ziemer & Rothmund, 2024). Debunking relies on professional fact-checkers to identify, label and correct misleading posts, aiming to decrease users’ belief in false information by alerting them to its inaccuracy. This type of intervention provides detailed explanations that disprove false information by replacing it with accurate facts after individuals have been exposed to the misinformation (Drolsbach et al., 2024). This can be time-intensive and may not completely counteract the effects of misinformation, such as the other available interventions. In practice, debunking efforts do not always reach those who need them most, as predisposed individuals to believe falsehoods often avoid corrective information (Hoes et al., 2024).

Social media platforms are promoting interventions, such as accountability by enabling real-time communication and feedback loops between governments and citizens, which reduces information asymmetry and builds public trust (Oye, 2025). They provide spaces for citizen oversight and participation, where individuals can question government decisions, expose irregularities, and mobilise collective action against corruption. These platforms also function as cost-effective tools for transparency, allowing the dissemination of policies, announcements, and progress reports directly to the public (Oye, 2025). At the same time, they create opportunities for two-way dialogue, ensuring that citizen concerns are acknowledged and integrated into governance processes, thereby reinforcing both transparency and accountability (Oye, 2025). Another important aspect of social platforms is the inclusion of advanced analytics and AI-driven real-time monitoring systems integrated into MDM platforms, which enable continuous anomaly detection, instant alerts, and automated corrective actions, making it possible to identify and counter the spread of misinformation as it emerges (Paul, 2025).

Another strategy is counter-messaging, which refers to truthful campaigns that counter disinformation by appealing to narratives and psychology rather than relying solely on factual correction (Bateman & Jackson, 2024). Research indicates that people are more receptive to messages aligned with their existing worldviews, especially when framed in moral or emotional terms and delivered by trusted in-group members perceived as respectful and acting in their interests (Wittenberg & Berinsky, 2020).

Despite the different types of interventions being implemented, attention must be paid to potential adverse effects. According to a recent study that analysed the negative effects of interventions against MDM, the authors concluded that interventions like media literacy tips and fact-checking may increase scepticism towards all kinds of information, true and false (Hoes et al., 2024).

The continuum of violent extremism frames radicalisation as a dynamic, non-linear process through which individuals move from ideological neutrality to potential engagement in extremist violence (McCauley & Moskalenko, 2017). This model allows for tailored prevention and countering violent extremism (P/CVE) interventions at various stages of ideological and behavioural development. In its early phase, individuals may be neutral yet vulnerable due to grievances or marginalisation. Here, misinformation, disinformation, and malinformation (MDM) can shape perceptions of identity, justice, and threat, increasing susceptibility to radical narratives (Harley & Cooper, 2021; Lewandowsky et al., 2012). Arguebly, preventive efforts should therefore prioritise media literacy, civic education, resilience to manipulation, and early detection, as the timing of detection is critical, as early identification can mitigate the adverse effects of rumour propagation (Bodaghi et al., 2023).

As individuals become sympathisers or justifiers - supporting extremist causes without endorsing violence - MDM reinforces in-group/out-group polarisation and legitimises violence through falsehoods and emotional manipulation. Counter-narratives, fact-checking, and community dialogue are likely important for disrupting these narratives (Morar & Santos, 2020). At advanced stages, where individuals adopt radical or terrorist behaviours, MDM often serves operational or propagandistic functions. At this point, security-based interventions are likely important, though digital strategies led by credible voices may support disengagement (Koehler, 2017).

Timing is critical: early-stage interventions, particularly with youth and marginalised communities, are more effective and resource-efficient, as beliefs are still forming (Hoferer et al., 2025). Promoting critical thinking and institutional trust reduces susceptibility to misinformation (Lewandowsky et al., 2012), while late-stage responses are riskier and less predictable. To effectively counter MDM across this continuum, P/CVE strategies must incorporate timely, multi-sectoral digital responses—monitoring harmful content, promoting inclusive narratives, and fostering collaboration with civil society and tech platforms. Doing so may pre-empt the deepening of ideological commitment and reduce the likelihood of violence.

### Why it is Important to do This Review

Despite the growing scientific literature in the field of MDM, there is a gap in the universe of publications, as no systematic review has analysed the interventions in this field to prevent and counter violent extremism. In fact, the research in this area is relatively new, with the first primary studies published in recent years.

Thus, the importance of this review is threefold. First, it is important for practitioners, as this review will demonstrate which types of MDM interventions reduce or prevent violent extremism, and potentially what factors might influence how effective an MDM intervention is (e.g., setting, types of populations, or intervention modalities). This is of paramount importance, as it is necessary to know the types of interventions that can reverse the impacts of MDM on extremism. Secondly, this review is important for policy-making, as evidence related to interventions on MDM to reduce and/or prevent violent extremism could shape future policies in this field and promote access to trustworthy, reliable and correct information, so that citizens can make informed decisions and live in a peaceful society. In addition, evidence of the effectiveness of interventions in this field can encourage policymakers and decision-makers to invest in evidence-based policies and practices. Thirdly, this review has relevance for research which then guides policy and practice. It is the first systematic review that will analyse how interventions on MDM can prevent or reduce violent extremism.The results of this review can guide future funding directions, particularly for projects and interventions aimed at investigating other pertinent aspects of how interventions on MDM can effectively prevent and counter violent extremism.

Despite the growing attention that MDM has been gaining in recent years, the information is still scattered and lacks synthesis. There is no systematic review that analyses the effectiveness of interventions on MDM to prevent violent extremism. Other systematic reviews have focused on different topics, including how to broadly counter rhetoric or narratives that may be rooted in simplified or misleading information (Carthy et al., 2020). In a review by Kapantai and colleagues (2020), these authors defined and characterised the underlying aspects and content types of the taxonomy of disinformation, providing useful directions for its definition. In another review, Valverde-Berrocoso et al. (2022) analysed some of the characteristics of the studies included in their review under this topic, such as geographical dispersion, and identified practices and literacy tools to overcome disinformation and misinformation. Beer and Matthee (2021) identified the main approaches used to identify fake news and how they can be applied in different situations. In another review, Di Domenico and colleagues (2021) proposed to analyse not only the definitions of fake news and how they spread, but also their consequences for consumers and companies. Ahmed and colleagues (2021) developed a review in which they analysed the role of and how machine learning may be used to detect fake news. Recently, a scoping review examined the psychological interventions to counter misinformation on social media. Its objectives were to identify and map evidence on psychological interventions to counter misinformation, to analyse the interventions on social media, and to produce insights for effective interventions (Gwiaździński et al., 2023). Similarly, a recent systematic scoping review analysed psychological interventions to hinder the dissemination and acceptance of misinformation, and the results demonstrate that most studies on misinformation focus on fact-checking interventions, but are weakly grounded in psychological theory and do little to address motivated reasoning (Ziemer & Rothmund, 2024). Another review organised and synthesised the field of user-centred misinformation interventions to promote knowledge sharing, highlight emerging trends, and inform evidence-based decision-making (Hartwig et al., 2024). The main conclusion highlights that transparent interventions - those that provide explanations and promote media literacy - are more effective than authoritarian approaches such as simple labelling or content removal.

Critically, while these reviews collectively establish the promise of MDM interventions across various contexts, none have explicitly examined their specific effectiveness in preventing or reducing violent extremism - a gap this systematic review addresses by consolidating evidence on how interventions targeting misinformation, disinformation, and malinformation can disrupt radicalisation pathways and counter violent extremist attitudes and behaviours.

In our preliminary exploration of relevant literature to potentially include in our systematic review, we identified some evaluative studies (Braddock, 2019; Carthy & Sarma, 2023; Lewandowsky & Yesilada, 2021). However, evidence is dispersed across various disciplines, and many concepts are employed interchangeably. We anticipate uncovering additional studies as we refine and broaden our search, particularly by incorporating grey literature. Given the interdisciplinary nature of the research landscape and the relative novelty of these interventions, particularly to prevent and counter extremism, conducting a systematic review is imperative. Such a review aims to scrutinise the diverse range of interventions being implemented, the outcomes under evaluation, and the overall quality and robustness of the evidence base. Given that interventions addressing the spread and acceptance of false and misleading information hold promise for mitigating violent extremism, transparent and evidence-based approaches to MDM can disrupt the mechanisms that contribute to radicalisation. The results of this review will be essential to effectively and efficiently inform policy and practice within the realm of this multidisciplinary intervention, specifically targeting its impact on violent extremist attitudes or behaviours. Moreover, access to diverse and reliable sources of information and, ultimately, a shared set of facts, is essential for well-functioning societies. This enables people to generate well-informed opinions, hold governments to account, and participate in public debate. Thus, this review will be a relevant contribution to inform policy-making through the production of critical scientific knowledge.

## Objectives

The research question that guides this review is: Which are the most effective interventions targeting MDM for reducing violent extremism? To respond to this research question, this review has the following objectives:

(1) To evaluate the effectiveness of interventions targeting MDM for reducing and/or countering violent extremist behaviour and attitudes.
(2) To examine whether intervention effectiveness varies according to the following moderators (a) study design; (b) type of population, (c) country in which the intervention took place; (d) theoretical framework of the intervention (e.g., inoculation theory); (e) stage of the intervention (prepare, curb, respond [see Johansson et al., 2022]); or (f) intervention characteristics (e.g., delivery mode, length of intervention, type of practitioner implementing the intervention).

## Methods

### Criteria for Considering Studies for This Review

No exclusions will be made based on year of publication, geographical location of the study or language. We will include studies irrespective of the manner through which they are published, as long as they refer to the analysis of primary data, either published or unpublished. Relevant reviews will be preserved for hand searching of their references.

#### Types of Studies

For studies to be considered eligible, they should include a target group where participants are exposed to an MDM intervention and a comparison group who are not. Studies that reflect the “natural” variations of said interventions will be eligible for inclusion. That is, where participants are exposed to naturally occurring MDM interventions, where there is no prospectively designed and structured intervention. To evaluate the effectiveness of MDM interventions, we will include experimental designs, with comparison group(s) receiving no intervention, business-as-usual, receiving the intervention at the end of the study period (waitlist control), or alternative interventions. Included designs are:

○ Randomised controlled trials (RCTs), in which participants are allocated to experimental or control groups using a predefined randomisation method (e.g., block randomisation, minimisation). Studies where the randomisation procedure is not clearly specified will also be included. We will accept designs with or without baseline measures. No restrictions will be made based on blinding, though potential bias related to blinding will be considered during the analysis.
○ Quasi-experimental designs, of any factorial design, where intervention and control groups are matched through validated methods (e.g., propensity score matching, coarsened exact matching, exact matching) or unmatched, as long as there is face validity or covariate adjustment in the analysis. Designs with or without baseline measurements of the outcome will be eligible for inclusion.

Protocols for prospective studies will be eligible for inclusion if they meet the inclusion criteria, and will be listed as an ongoing study in the respective reference list.

Interventions are highly context-dependent, shaped by the characteristics of specific actors and settings, and are thus difficult to replicate (Hirschi & Widmer, 2012). Evaluation approaches must remain flexible and tailored to the intervention in question, yet this adaptability contributes to a proliferation of methods and a lack of standardisation. Where definitions of core terms diverge at the intervention level, evaluators must determine their applicability for analytical purposes.

Studies that focus on the economic evaluation or cost-effectiveness of interventions will not be eligible for inclusion.

#### Types of Participants

This review will focus on people who are on the receiving end of information that falls in the spectrum of MDM, as previously defined. Participants of included studies do not necessarily need to be at-risk of or have shown violent extremist behaviours and/or attitudes. No exclusions will be made based on characteristics of the population, including but not limited to gender and/or sex, ethnicity, and age.

#### Types of Interventions

Eligible studies should report the evaluation of an intervention in which the primary component is aimed at countering and/or reducing the effects of MDM. As such, any intervention with strategies, activities, training, and/or other techniques to reduce the effect of MDM will be eligible for inclusion, though no exclusions will be made based on the main purpose of the overall intervention. As described in the ‘How the intervention might work’ section, example intervention approaches include debunking, fact-checking, and digital literacy models. Based on a preliminary review of the literature, many eligible interventions are likely to be underpinned by inoculation theory (McGuire, 1961), which posits that individuals are notified of an upcoming persuasive attempt (explicit forewarning) to stimulate counter-arguing (refutation), thereby enhancing their resistance. Example interventions that would be eligible for this review include:

(1) Braddock’s (2019) attitudinal inoculation study, where participants received an online intervention where they were explicitly forewarned that they would be exposed to MDM before exposure (i.e., inoculation). This intervention sought to increase resilience on MDM present in both extreme left and right propaganda, through an experimental design with two no-inoculation control conditions (extremist left-wing vs. extremist right-wing) and two inoculation conditions (researcher vs. former extremist).
(2) Lewandowsky and Yesilada’s (2021) attitudinal Inoculation study which was built on the premise that psychological inoculation protects against misleading rhetoric. The intervention was delivered to United Kingdom residents recruited via Prolific. After a careful analysis of YouTube content on Islamophobic and radical-Islamist content, the authors used an experimental design in which they tested no intervention vs. inoculation and Islamophobic misinformation vs. radical-Islamist misinformation. The allocation of participants to one of the four groups was randomly implemented, after which they watched a video on the training material when assigned to the intervention (inoculation condition), or unrelated material if they were in the control group. Depending on the group, participants would then be exposed to radical-Islamist or Islamophobic content.
(3) Carthy and Sarma’s (2023) counter-arguments study, where participants were recruited at an Irish university and which used an experimental design with randomisation to one of four conditions. Participants were either provided with counter-arguments about incorrect information or instructed to create their own counter-arguments (inoculation) at regular intervals while being exposed to MDM.

#### Types of Outcome Measures

Included studies must report the effects of the MDM intervention on attitudinal or behavioural measures of violent extremism. Whereas attitudinal indicators provide insight into ideological shifts and willingness, they are considered intermediate or proximal, and thus less directly indicative of real-world threat; behavioural indicators, though rarer and more difficult to assess, serve as ultimate outcomes for intervention evaluations (Borum, 2015; Moskalenko & McCauley, 2009; Scarcella et al., 2016; Wolfowicz et al., 2021).

In the context of violent extremism, attitudinal measures can be defined as psychological constructs reflecting the degree to which an individual expresses support for, justification of, or personal obligation toward violent acts (Ajzen & Fishbein, 1980; McCauley & Moskalenko, 2017; Wolfowicz et al., 2021). These measures are typically assessed using validated psychometric instruments or scales such as the Extremism Scale (ES) and the Pro-Violence and Illegal Acts in Relation to Extremism Scale (PIARES), which capture both the endorsement of extremist beliefs and the acceptance or justification of violence and illegal means in the service of an ideology (Ozer & Bertelsen, 2018). Attitudinal measures may include items that probe willingness to engage in violence, perceived legitimacy of using violent means, or degree of identification with extremist groups and their cause (Moskalenko & McCauley, 2009; Doosje et al., 2016). These are often operationalised through self-report questionnaires, but may also be coded quantitatively from semi-structured interviews or narrative data (Clemmow, Rottweiler, & Gill, 2022). For the review, attitudinal outcomes are understood as indicators of cognitive radicalisation, and although they may represent precursors or correlates to behavioural radicalisation, not all individuals endorsing such attitudes proceed to action (Schmid, 2013; Borum, 2011; Wolfowicz et al., 2021).

Behavioural measures, by contrast, are concerned with observable acts or externally verifiable activities that are ideologically motivated and meet the definitional criteria of violent extremism (Schmid, 2013). These can include formally documented violent actions, such as completed or attempted attacks, direct incitement to violence, or other offences resulting in arrest, prosecution, or conviction (Scarcella et al., 2016). Behavioural indicators also extend to verified involvement in the planning or threat of attacks, active membership in violent extremist organisations, or participation in preparatory acts such as stockpiling weapons, arranging logistics, or materially supporting terrorist activity (Pressman & Flockton, 2012). Sources of behavioural measures often include official records from law enforcement, judicial sentences, corrections data, or third-party verification by programme staff (Scarcella et al., 2016; Wolfowicz et al., 2021). These outcomes are viewed as the end-stage manifestation of radicalisation processes, and are distinct from, though related to, cognitive and affective antecedents (Borum, 2015).

#### Duration of Follow-Up

No restrictions will be made based on the duration of follow-up, as we will include outcomes measured at post-intervention and any follow-up time point.

#### Types of Settings

No limitations will be imposed pertaining to the settings of the interventions.

### Search Methods for Identification of Studies

#### Electronic Databases

A comprehensive search that includes multiple electronic academic databases and grey literature repositories will be used to gather published and unpublished evidence. This strategy aims to reduce publication bias and provide a balanced representation of existing evidence (Paez, 2017). We sought to assess and elicit relevant terminology on the topic through a preliminary search on APA PsycINFO (via EBSCO) and Criminal Justice Abstracts (via EBSCO). After careful analysis of titles, abstracts and keywords from relevant papers, we defined a search query with three categories: (1) topic of the review, (2) intervention, and (3) outcomes, with terms combined with the Boolean operator OR and the categories with AND. The template search structure is presented below in Table 1 with a fully executed search for APA PsycINFO (via Ovid) provided in Appendix 1. The complete list of electronic databases can be consulted in Table 2.

**Table 1.** Search Terms

Line	Search terms	Search fields
1	(disinformation* or “fake news” or malinformation* or misinformation* or misperception* or mistruth* or propaganda*)	Title, Abstract, Author-Supplied Keywords, Subject/Indexing Terms
2	(corrected or correcting or correction* or counter* or debunk* or deplatform* or de-platform* or detect* or disprov* or educat* or “fact check” or “fact checking” or “fact checked” or “fact checks” or “fact-check” or “fact-checking” or “fact-checked” or “fact-checks” or gamif* or inoculat* or “literacy training” or “media literacy” or persuad* or persuas* or prebunk* or prevent*)	Title, Abstract, Author-Supplied Keywords, Subject/Indexing Terms
3	(randomi* or randomly or rct* or allocat* or assign* or “between subjects” or blind or blinded or “control group” or “controlled study” or experiment* or “factorial design” or group or groups or interven* or matched or nonrandom* or non-random* or “non random” or “non randomised” or “non randomized” or “non randomly” or “open label” or open-label or placebo* or “pre-post” or quasiexperiment* or quasi-experiment* or quasirandom* or quasi-random* or trial* or “within subjects”)	Title, Abstract, Author-Supplied Keywords, Subject/Indexing Terms
4	1 AND 2 AND 3	​

**Table 2.** Search Resources

Source type	Source/URL
Electronic Databases	Ovid: - APA PsycINFO ProQuest: - APA PsycArticles - Applied Social Sciences Index & Abstracts (ASSIA) - Dissertations & Theses Global - Educational Resources Information Center (ERIC) - International Bibliography of the Social Sciences (IBSS) - Social Services Abstracts - Sociological Abstracts Web of Science: - Book Citation Index - Social Sciences & Humanities (BCI) - Conference Proceedings Citation Index - Social Sciences & Humanities (CPCI-SSH) - Emerging Sources Citation Index (ESCI) - SciELO Citation Index - Social Science Citation Index (SSCI) EBSCO: - Criminal Justice Abstracts (CJA) - International Political Science Abstracts (IPSA) Informit: - Australian Criminology Database (CINCH)
Institution/Organization	
Digital Public Square	https://www.digitalpublicsquare.org/
Royal United Services Institute	https://rusi.org/
Center on Hate, Bias and Extremism (OntarioTech University)	https://socialscienceandhumanities.ontariotechu.ca/centre-on-hate-bias-and-extremism/index.php
Center for Information Integrity (University of Buffalo)	https://www.buffalo.edu/cii.html
Center for International Governance Innovation	https://www.cigionline.org/
Center for Strategic and International Studies	https://www.csis.org/
Commission for Countering Extremism	https://www.gov.uk/government/organisations/commission-for-countering-extremism
Council of Europe	https://www.coe.int/en/web/portal
Crime and Security Research Institute (Cardiff’s University)	https://www.cardiff.ac.uk/security-crime-intelligence-innovation-institute
Department of Homeland Security	https://www.dhs.gov/
European Foundation for South Asian Studies	https://www.efsas.org/
Institute for Strategic Dialogue	https://www.isdglobal.org/
International Centre for Counter-Terrorism	https://www.icct.nl/
Home Office, UK (including Ministry of Justice)	https://www.gov.uk/government/organisations/home-office
Jill Dando Institute of Security and CrimeScience	https://www.ucl.ac.uk/jill-dando-institute
NATO	https://www.nato.int/
RAND Corporation	https://www.rand.org/
The Senator George J. Mitchell Institute for Global Peace, Security and Justice (Queen’s University Belfast)	https://www.qub.ac.uk/Research/GRI/mitchell-institute/
United Nations Interregional Crime and Justice Research Institute	https://unicri.it/
DART-Europe E-theses Portal	https://www.dart-europe.org/basic-search.php
Networked Digital Library of Theses and Dissertations	https://ndltd.org
Journal	Critical Studies on Terrorism; Dynamics of Asymmetric Conflict; Group Processes and Intergroup Relations; Information Systems Journal; Intelligence and Counter Terrorism; International Journal of Conflict and Violence; Journal for Deradicalization Journal of Information Literacy; Journal of Interpersonal Violence; Journal of Policing; Journal of Politics; Perspectives on Terrorism; Police Quarterly; Policing—An international Journal of Police Strategies and Management; Policing and Society; Political Communication; PNAS Nexus; Sciences of Terrorism and Political Aggression; Studies in Conflict & Terrorism; Terrorism and Political Violence.

#### Searching Other Sources

In order to locate unpublished studies or literature that cannot be found in academic databases, other sources will be consulted. These sources correspond to think tanks, international organisations, university research centres and civil society organisations (see Table 2).

Publications from selected journals that are published six months before the execution of the systematic search will be analysed to identify potentially eligible studies that have not yet been indexed in academic databases. The list of journals for hand-searching can be consulted in Table 2.

Reviews retrieved and/or identified during the search will be preserved for harvesting of potentially relevant studies from their reference lists. Experts on the field will also be consulted for relevant material on the field, particularly authors of eligible studies, in order to retrieve unpublished studies, studies in development, or studies that were missed during the search process (Young & Hopewell, 2011). Following previously defined eligibility criteria, we will use included studies to conduct backward citation searching through a hand search of their references, and forward citation on Google Scholar to identify potentially eligible studies, published or unpublished, that have not been captured by the systematic search.

The retrieval process will be documented with enough detail to fill the information from the PRISMA flow chart (Page et al., 2021) and to produce a full search history for the final review.

### Data Collection and Analysis

#### Description of Methods Used in Primary Research

Based on preliminary searches, we expect included studies to be mostly experimental and then quasi-experimental designs, with differences being registered in the target population of the intervention, methodological differences in the outcome measurement, but mostly grounded in inoculation theory. In terms of participants, the preliminary search showed that participants are usually adults (i.e., older than 18 years old). Some are recruited online, while others are recruited in universities (e.g., Carthy & Sarma, 2023; Lewandowsky & Yesilada, 2021). It is expected that most outcomes will vary between violent extremist behaviour (attacks, inciting to violence, threat of attacks, sentences, or convictions) and violent extremist attitudes (willingness to perform violent extremist behaviour). In terms of design, many studies that use inoculation theory are experimental, namely the one conducted by Braddock (2019) where participants were randomly assigned to each group and some received the inoculation messages. In this study, in the inoculation conditions, participants received the forewarning that one of the messages came from a “political extremist group”. Only participants identified with white ethnicity were assigned to the right-wing condition, as it was expected that non-white participants would feel an immediate aversion to messages whose content related to white supremacy and purity. After exposure to propaganda, participants were asked to complete a survey measuring counter-arguing, feelings of anger, behavioural intentions, source credibility and social dominance orientation.

#### Selection of Studies

This review is linked with another review with similar author teams (Duarte et al., 2025). The searches for these reviews have been combined and de-duplicated in EndNote, and the study selection process will occur for both of these reviews simultaneously. Through EPPI-Reviewer (Thomas et al., 2023), the title/abstract screening phase will focus on excluding studies that are not related to violent extremism (and related terms), with every study being screened by two independent reviewers, as a means to increase the number of relevant records identified at this phase (Stoll et al., 2019). Discrepancies between reviewers will be identified and solved through discussion until consensus is achieved. If consensus cannot be reached, a third reviewer will be consulted. Studies retained at this initial screening stage will then be appraised for relevance to each respective review, based on full-text documents. Authors of studies with unavailable full-texts will be contacted to facilitate access. Full-text documents will be screened by two independent reviewers, and for every exclusion, one of the following reasons will be attributed: (1) study not available; (2) empirical study not on MDM; (3) ineligible study design; and (4) ineligible outcome measures. Any conflicts that arise from this phase will be discussed between all authors until consensus is achieved. Studies screened as unavailable will be listed in the ‘Studies awaiting classification’ reference list in the final review.

#### Data Extraction and Management

Data from studies considered eligible for inclusion will be independently coded by two reviewers according to a data extraction form (see Appendix 2). Extracted information will then be compared and collated into a final form for synthesis and analysis. Discrepancies or disagreements between coders will be resolved through discussion and consensus. If consensus cannot be reached, a third reviewer will be consulted to mediate and make a final decision. The primary categories for extraction will be:

● General information (i.e., publication type, funding details);
● Study characteristics (i.e., study and/or factorial design);
● Participant characteristics (i.e., recruitment method, eligibility criteria, sample size, sample features, baseline imbalances, withdrawals/exclusions);
● Intervention (i.e., allocation, setting, theoretical framework, description, duration, provider characteristics, cointerventions, delivery mechanism, compensation, cost of intervention);
● Outcome (i.e., time points measures, follow-up, measurement, validity, reliability, upper and lower limit, thresholds, interpretation, missing data, power, statistical significance, direction of outcome, group favoured, main conclusions);
● Effect size (i.e., total sample sizes, attrition, raw effect size, measure value).

#### Measures of Effect

The structured coding form includes built-in sections that enable two independent coders to extract raw data from the included studies, such as means, standard deviations, sample sizes, and/or proportions. Depending on the measures used and how the authors choose to treat the raw data, outcomes related to violent extremist behaviours and/or attitudes can be reported as either continuous (e.g., mean number of violent acts over a given time period) or binary (e.g., proportion of participants who have committed violent acts).

We propose using logarithmic Odds Ratios (logOR) as the primary metric for outcome measures, along with the corresponding 95% and 90% confidence intervals (95%/90% CI). logOR values significantly greater than 0 will indicate a desirable impact of the MDM intervention on measured outcomes, while values significantly less than 0 will indicate an undesirable impact. A logOR value of 0 will represent a null intervention effect.

For outcomes reported as continuous, Cohen’s *d* will serve as the primary metric, paired with the corresponding confidence intervals 95% CI. Cohen’s *d* will then be transformed into logOR using Cox and Snell’s approach, which assumes a normal distribution and equal variances between groups (Anzures-Cabrera et al., 2011). This transformation will be achieved by multiplying Cohen’s *d* and its standard error by 1.65.

#### Assessment of Risk of Bias

Risk of bias will be assessed using the tools provided by the Cochrane Collaboration. The Risk of Bias tool (RoB (2) will be used for randomised trials (Sterne et al., 2019). RoB 2 provides a framework for critical assessment based on five domains: (1) randomisation process; (2) deviations from intended interventions; (3) missing outcome data; (4) outcome measurement; and (5) selection in the reported results (Sterne et al., 2019). Overall bias is, thereafter, stratified as “high”, “low” and “some concerns” (Sterne et al., 2019).

The Risk Of Bias In Non-randomised Studies of Interventions (ROBINS-I) tool will be used for non-randomised studies (Sterne et al., 2016). This tool is structured in three domains, specifically: (1) pre-intervention, referring to confounding and participants’ selection; (2) intervention focusing on bias arising from the classification of the interventions; (3) post-intervention, entailing bias due to deviations from intended interventions, missing data, outcome measurement and selection in the reported results (Sterne et al., 2016). Domain-level and overall risk of bias in ROBINS-I has four different levels, i.e., “low”, “moderate”, “serious”, and “critical”, complemented with a no information option, for when the existing data is not sufficient to support an appropriate judgement (Sterne et al., 2016).

Included studies will be independently assessed by two members of the research team according to the rules of each tool. Any discrepancies will be resolved through consensus. If consensus cannot be reached, a third member of the research team will be consulted. Authors of studies that remain coded as “unclear” will be approached as a means to gather information to fill existing gaps. Risk of bias across and within studies will be presented in a summary table and figure, respectively. No studies will be excluded solely based on the risk of bias assessment. However, the potential influence of bias on review findings will be formally examined through planned sensitivity analyses, in which studies at high or critical risk of bias will be excluded to assess the robustness of the results. The outcomes of these analyses will be reported and discussed in the final review to ensure transparency in interpreting the evidence.

#### Unit of Analysis Issues

Clustering is one potential unit of analysis issue anticipated for this review. Clustering adjustments will be applied only to studies in which allocation or delivery occurs at the cluster level (e.g., universities, communities, group versus individually delivered interventions) and individual-level outcomes are analysed without an appropriate cluster adjustment. We will seek to obtain an estimate of the intracluster (or intraclass) correlation coefficient (ICC), based on the following hierarchy: (1) use an ICC reported in the study; (2) if unavailable, request the ICC (or cluster-adjusted results) from study authors; or (3) if still unavailable, use an external ICC drawn from methodologically similar populations, settings, and outcomes (Higgins et al., 2019).

A primary unit of analysis issue relates to studies randomising participants to multiple eligible intervention groups. Rather than applying ad hoc solutions to individual problems, we adopt a unified analytical framework guided by López-López and colleagues (2018), which systematically identifies types of effect size multiplicity and defines appropriate statistical solutions.

Many studies generate multiple effect sizes through (1) multiple eligible intervention arms, (2) outcomes measured at multiple follow-up timepoints, or (3) a combination thereof. These effect sizes are non-independent within studies. We will handle such dependencies using robust variance estimation (RVE) with correlated effects meta-analysis, which accommodates all non-independent effect sizes within a single model without loss of information (Pustejovsky & Tipton, 2022). This approach is conducted using the *robumeta* package in R and simultaneously addresses clustering from both multi-arm designs and repeated measurement. Under the RVE approach, we include: (1) all eligible intervention arms (rather than merging substantively different interventions); (2) all prespecified follow-up timepoints (rather than selecting one timepoint per study); (3) effect sizes from all arms compared to a common reference (typically a merged control group), allowing pairwise treatment comparisons when required.

Effect sizes will be extracted separately for each relevant timepoint and intervention arm comparison. While effect sizes will be extracted and coded separately by timepoint for organizational clarity, the RVE framework subsequently models their dependency structure rather than treating each timepoint grouping as an independent analysis, thereby accounting for correlation within studies. Where reporting intervals do not align precisely with our prespecified groupings (immediate post-intervention, short-term follow-up, long-term follow-up), the timepoint closest to the relevant grouping will be selected.

Should preliminary analyses reveal excessive heterogeneity or if substantive research questions require comparison of distinct intervention types (e.g., face-to-face vs. online delivery), we may conduct separate RVE models for predefined intervention subsets. Any such subgroup analyses will continue to employ the RVE framework; this does not involve reduction to single effect sizes per study, and the within-study dependency structure is preserved across all subgroup comparisons. This approach partitions the evidence base meaningfully while maintaining statistical rigor and avoiding information loss.

#### Criteria for Determination of Independent Findings

Salami and dependent publications are mechanisms through which dependent findings might be uncovered, either through the fragmentation of data into the smallest units for publication or the publication of identical or similar results across multiple papers (Ding et al., 2020). In the scenario where included studies are signalled as being based on the same data set, resulting in two or more publications, results from these studies will be collated and reported on a single coding form. Determining dependence will be based on the detection heuristic proposed by Woods (2007), taking into account methodological dimensions such as study and sample characteristics, authorship details, constructs and measures, as well as effect sizes. Where multiple measures of the same construct are available for the same analytic timepoint, handling will be guided by López-López et al. (2018), prioritising a single effect size per construct per timepoint or applying appropriate aggregation methods. Grouping by follow-up time and decisions regarding repeated outcome measurement are addressed separately in Section 3.3.6.

#### Dealing With Missing Data

If studies do not report data deemed essential to compute effect sizes, the corresponding authors of said studies will be contacted to provide the required missing data. If authors are unable to provide missing data or cannot be contacted, other data will be used to calculate effect sizes wherever possible (e.g., p-values and t-scores). If neither of these options permits calculation of effect sizes, the study will be excluded from the meta-analysis, but will be included in the description of the included studies and risk of bias assessment.

#### Assessment of Heterogeneity

We will provide a narrative summary of the differences between studies based on their design and PICO (Population, Intervention, Comparator, and Outcome) dimensions to illustrate methodological variations across those included. To formally assess the variance in effect sizes and the degree of heterogeneity, we will employ two key statistical measures: Cochran’s Q, *I*^2^ and *τ*^2^. Cochran’s Q evaluates whether the observed variability in effect sizes exceeds what would be expected due to sampling error alone. However, this metric is sensitive to the number of included studies and may fail to detect meaningful heterogeneity (Ionnaidies et al., 2007). *I*^2^ quantifies the proportion of total variability in effect sizes that is attributable to heterogeneity rather than chance, by providing a standardised measure for comparing heterogeneity (Higgins et al., 2003). *τ*^2^ estimates the between-study variance in effect sizes on the original scale of measurement, indicating the magnitude of variation in effect sizes across studies (Higgins et al., 2019). Formal statistical analyses of heterogeneity will be conducted using the metafor package in R, with the dmetar package assisting in reporting and visualisation.

#### Assessment of Reporting Biases

Publication bias and small-study effects will be examined using regression-based methods compatible with robust variance estimation (RVE). Small-study effects will be assessed using precision-effect test and precision-effect estimate with standard error (PET-PEESE) meta-regression models, in which effect sizes are regressed on their standard errors (PET) and on their variances (PEESE), fitted within an RVE framework to account for dependent effect sizes. Egger’s regression test for funnel-plot asymmetry will also be implemented using the same RVE structure, by regressing standardized effect sizes on their precision. All meta-analytic models, including PET-PEESE and Egger-type regressions, will be fitted in R using the metaphor and the clubSandwich package.

#### Data Synthesis

If possible, a meta-analysis will be conducted to assess the effectiveness of MDM interventions, provided that at least two studies are available for each conceptual outcome grouping. A random-effects model will be employed to account for variability between studies and to provide more generalised estimates of intervention effectiveness. The results of the meta-analyses will be summarised in forest plots, which will display pooled effect estimates alongside their 95%/90% CI, allowing a comparison of results across studies.

Meta-analyses will be performed using the metafor package in R for model fitting and estimation, supported by the dmetar package for visualisation and supplementary analyses. Studies will only be withheld from pooling when they are conceptually unique, that is, when outcomes, populations, interventions, or measurement scales cannot be operationally grouped with others in a defensible way (e.g., unique constructs or incompatible measurement frameworks). In such cases, we will report standardised individual effect sizes and provide a structured narrative synthesis to preserve transparency while acknowledging non-comparability.

#### Subgroup Analysis and Investigation of Heterogeneity

Moderator effects will be examined exclusively using meta-regression models to evaluate whether the effects of MDM interventions vary according to key study or intervention characteristics. This approach is preferred to stratified meta-analysis and avoids the reduction in statistical power and instability associated with estimating outcome parameters in small subgroups (Higgins et al., 2019, pp. 265-72). Moderators of interest, including intervention characteristics, theoretical framework (e.g., inoculation theory), intervention stage, study setting, study population, length of follow-up, study design, and risk of bias, will be explored via meta-regression, contingent on having at least ten studies for each moderator. All moderator and heterogeneity analyses will be conducted using the metafor package in R, with additional reporting and graphical functions from dmetar as needed.

#### Sensitivity Analysis

Assessing the impact of decisions made throughout the review process is crucial for understanding their potential influence on the meta-analysis results. Some decisions may be arbitrary, such as defining threshold values for certain parameters, or ambiguous due to insufficient transparency in the reporting of methods in primary studies. Sensitivity analysis plays a key role in evaluating the robustness of the review findings by determining whether the results remain consistent despite these decisions. To achieve this, we will run new versions of the meta-analysis to examine the impact of such decisions on the pooled effect estimates by removing studies identified as having a high or critical risk of bias. The results of the sensitivity analysis will be presented in a summary table, following the recommendations outlined by Higgins et al. (2019).

#### Treatment of Qualitative Research

The present review will not include studies using qualitative designs.

## Supplemental Material

Supplemental Material - Interventions Targeting Misinformation, Disinformation and Malinformation for Reducing and Countering Violent Extremism: A Systematic Review Supplemental Material for Interventions Targeting Misinformation, Disinformation and Malinformation for Reducing and Countering Violent Extremism: A Systematic Review by Cátia Moreira de Carvalho, João Pedro Ramos, Catarina Simas, Pedro Barbosa, Sarah Carthy, Marta Pinto in Campbell Systematic Reviews

Supplemental Material - Interventions Targeting Misinformation, Disinformation and Malinformation for Reducing and Countering Violent Extremism: A Systematic Review Supplemental Material for Interventions Targeting Misinformation, Disinformation and Malinformation for Reducing and Countering Violent Extremism: A Systematic Review by Cátia Moreira de Carvalho, João Pedro Ramos, Catarina Simas, Pedro Barbosa, Sarah Carthy, Marta Pinto in Campbell Systematic Reviews
