# Supplementary material for: PROTOCOL: Interventions Targeting Misinformation, Disinformation and Malinformation for Reducing and Countering Violent Extremism: A Systematic Review
Source: Campbell Syst Rev. 2026 Jun 21;22(2):18911803261459099. doi: 10.1177/18911803261459099 (PMC13305521; doi:10.1177/18911803261459099)
Supplement: Supplemental Material - Interventions Targeting Misinformation, Disinformation and Malinformation for Reducing and Countering Violent Extremism: A Systematic Review [file sj-pdf-1-cam-10.1177_18911803261459099.pdf]

Key to Reviewers = ED: Editor; AE: Associate Editor; ME: Methods Editor; IR: Information Retrieval specialist; R1: Reviewer 1; R2: Reviewer 2.

Consolidated reviews: Interventions targeting misinformation, disinformation and malinformation for reducing and countering violent extremism [PROTOCOL]

Campbell Collaboration Crime and Justice Coordinating Group

General points Only	Feedback	Reviewer	Revised?	Author comments	ED Use
1. General Points	In general this is a respectable scientific approach to the problem that seems somewhat out of touch with many of the current findings and understanding in the MDM domain.	R1	x	Thank you. We have carefully revised our protocol according to revisions made.	☐
2. General Points	The language is understandable and suitable, with only a few minor errors in spelling or typing.<br>**Editorial comment:** Please ensure your manuscript has had a thorough proofing before resubmission.	R2	x	Thank you. We carefully revised our protocol before submitting to ensure there are no spelling or typing errors.	☐
3. General Points	A slight note before sharing my feedback: my comments are rather critical, but I hope that my detailed observations and concrete suggestions will be helpful in strengthening the manuscript and refining its focus.<br><br>Overall, I believe the manuscript does not do a good job of justifying the need for a systematic review. There are bits and pieces all over the manuscript that could be much better connected to each other. For instance, I believe the whole setup would be much stronger if the authors would start by highlighting that there is continued and even increased worry about the impact of MDM, especially the degree to which MDM can reinforce or even spark violent extremism (and lead with real-world examples here, if any). This is the case, even despite strong empirical evidence on its impact (e.g., are these events truly sparked by MDM?).<br><br>Regardless of the evidence, concerns about the	R3	x	Many thanks for your helpful comments. We have re-drafted several sections to address the cohesion issue better.<br>● In Section 1 (Background), we now place greater emphasis on why MDM poses a greater societal threat, drawing from the health literature before landing on the radicalisation literature. Here, we are establishing early on that MDM in the context of extremism presents a novel, unprecedented threat. On Section 1.1. On the "lengthy theoretical definitions" aspect, we got rid of the detailed description of the two pyramids model and defined radicalisation and extremism in a more coherent way.<br>● We have also provided a more informed synopsis (drawing from the wider radicalisation and communication literature) on the degree to which MDM can reinforce (or "spark") violent extremism. Here, we	☐

	impact of MDM have manifested to such an extent that dozens of interventions have been designed at all levels of society, including at the governmental level. For example, how many fact-checking organizations have surged since 2016? A lot! The authors could make their case much stronger by addressing these trends. However, the effectiveness of these interventions remains unclear, complicating the development of coherent, evidence-based approaches at higher levels of society. A review is needed to provide a comprehensive overview of the effectiveness of interventions to inform adequate policy measures. The current manuscript does not communicate this need well at all and, in my view, therefore needs to be revised quite dramatically, leaving out lengthy theoretical definitions and strengthening a clear-cut practical focus on MDM's real-world impacts and interventions.			also use some real-world examples (e.g., Edgar Maddison Welch).	
4. General Points	**Scope of MDM**: The manuscript does not acknowledge anything about the actual scope of misinformation. How much of it, as far as we know, is actually out there? How often do people consume or encounter it? Including these figures would provide crucial context for evaluating the relevance and effectiveness of interventions.	R3	×	We have addressed this point in several ways throughout the protocol.<br>● In Section 1 (Background), we speak more about the scope of misinformation and mention actions taken by social media companies.<br>● In Section 1 (Background) we also place greater emphasis on *why* MDM poses a greater societal threat, drawing from the health literature (where it is already described as an "infodemic") before introducing the radicalisation literature where the phenomenon is amplified by both scale and, notably, manipulation by external actors (i.e., violent extremist organisations).	☐

5.	General Points	**Intent and Intervention**: The authors talk about MDM, but the difference between these concepts is not meaningfully used in the manuscript. To what extent does intent matter for interventions? Intent is very hard and often impossible to establish, so are different interventions appropriate depending on intent, or should all false information, regardless of intent, be met with the same interventions? The authors do not engage with this at all, but in my view, they should. Additionally, which interventions are most effective online, and which offline? Since there is a whole paragraph devoted to governmental measures, which interventions are most relevant for them? This discussion is entirely missing.	R3	×	We have now incorporated additional information on interventions; however, we have not addressed their effectiveness, as this is the primary aim of the review. Consequently, the question "Which interventions are most effective online, and which offline?" will be addressed in the discussion section of the completed review, rather than in the protocol.	☐
6.	General Points	**Structure and Clarity**: The order of paragraphs is confusing. What is discussed in each paragraph is a surprise, as it does not follow an intuitive order. The paragraphs themselves are also sometimes poorly structured, and the writing style does not help—it's not clear or concise and jumps from one thing to another.	R3	×	Upon reviewing this feedback, we agree that our structuring of paragraphs was poor. We have completely re-drafted much of the manuscript, including:<br>● In Section 1 (Background), we now introduce the phenomenon in a more gradual way, drawing from the health literature (and presenting some behavioural implications of misinformation) before introducing the radicalisation literature. Notably, we try to 'scene-set' by including a real-life example early on (i.e., "pizzagate").<br>● Section 1.1. now deals exclusively with the relationship between MDM and violent extremism, rather than the long, theoretical discussions on radicalisation itself.	☐
7.	Abstract	The abstract reads alarmistic, particularly with the claim that MDM "have been linked to an	R3	×	Thank you. To fully address this point we have re-	☐

	unprecedented societal impact", which lacks empirical evidence. Contribution is framed as twofold: interventions designed to counter or reduce the effects of exposure to false information, and their impact on violent extremism (violent extremist behavior and attitudes). However, later in the abstract, this is reframed as "reducing susceptibility to false narratives," which is a different focus altogether. The abstract should be tailored more closely to the actual content of the review, and the tone should align with what we empirically know about MDM effects—far less alarmist than implied here.			drafted the abstract. We have removed the alarmist language and simply spoken about speed, scale and volume as well as the emergence of multiple methods of information transmission, all of which we provide evidence for in Section 1 (Background). We also removed "reducing susceptibility to false narratives" and kept the focus on reducing and/or countering.	

Background Only	Feedback	Reviewer	Revised?	Author comments	ED Use
8. Description of the condition	In the background, it is mentioned that the dissemination of false information is not a new phenomenon but has reached a new threshold in recent years. However, the reasons or factors that have intensified this problem should be explicitly mentioned.	R1	×	Thank you. In Section 1 (Background) we now place greater emphasis on *why* MDM poses a greater societal threat, arguing that the phenomenon is amplified by both scale (i.e., speed), the elicitation of emotions and, notably, the potential for manipulation by external actors. This is what distinguishes MDM in the context of radicalisation from historical manifestations.	☐
9. Description of the condition	"By adopting the typology proposed…" (p.3). can raise this question that why are you adopting this typology. To provide conceptual clarity/consistency/nuance that is often lacking? You can refer to the recent published review papers such as "A literature review on detecting, verifying, and mitigating online misinformation. (2023)", which provides the same typology for various types of false information.	R1	×	Thank you. We included that reference and clarified that the distinction is to provide conceptual clarity.	☐
10. Description of	"The most frequent topics spread seem to be those of health, politics, and disaster (Muhammed &	R1	×	Thank you. In Section 1 (Background) we have removed this citation and now place greater	☐

the condition	Mathew, 2022) and this may cause confusion and mislead people about important decisions about their lives and society" (Adjin-Tettey, 2022).(p.3). What do you mean by "decisions about society"? This fails to provide a compelling case for this review/seems to lack any sense of urgency. I would suggest adding something along these lines "…as information integrity is crucial for democratic processes and protecting fundamental rights. When information is manipulated, it can incite violence and discrimination, and authoritarian regimes often exploit digital technology to undermine trust in democracy (Government of Canada, n/d)." (p. 7) to the first paragraph.			emphasis on *why* MDM poses a greater societal threat:<br>● First, we draw from the health literature where we mention its effects on health behaviours.<br>● Next, we talk about its effect on other behaviours, including violent extremism. To elucidate this point, we provide a real-world example (i.e., "pizzagate").<br><br>In Section 1.1, we also connect exposure to MDM to a range of radicalisation behavioural outcomes.<br><br>We also removed any references to information integrity in order to make the paper more congruent and avoid concepts that might cause confusion to the reader.	
11. Description of the condition	In the section on "Actions to prevent and/or counter MDM", you briefly discussed actions to tackle, prevent, mitigate, and verify MDM through fact-checking. However, another crucial aspect_ early detection_ has been overlooked. It would be beneficial to include this aspect to provide a more comprehensive approach.	R1	×	Thank you for your suggestion. Given that we discuss the timeframe of interventions in Section1.4., we mention early detection there.	☐
12. Description of the condition	A comparison of MDM to other related concepts such as "prejudges", "stereotypes" including historical phenomena such as antisemitism or Islamophobia is missing. For example, if "misinformation as deceiving information disseminated without the intention of manipulating or causing harm; disinformation as the deliberate use of erroneous information to manipulate, cause harm or confuse people; and malinformation as the dissemination of correct information out of context	R1	×	Thank you. We have addressed this comment by expanding our paragraph on definitions, distinguishing between the three concepts in Section 1 (Background) using theoretical lenses rather than simple operationalisations.<br>● First, we propose that the three concepts are distinguished from each other on two grounds: truth and intention.<br>● We explain that while the intent differs, the processing of misinformation and	☐

	with the intent to cause harm, manipulate and confuse" (p.2), then (1) how does this definition differ or relate to other (e.g., social, community-based, historical) forms of disseminating harmful information as well as (2) how does it differ or relate to other definitions or understandings (within psychology, sociology, anthropology) of harmful/misleading knowledge or information, such as stereotypes, prejudices, racism, propaganda, or even Galtung's understanding of cultural violence. Also, is the context, vehicle/medium, environment and audience the key factor for how MDM is understood or conceptualized today, and how can we apply our modern understanding of MDM(*) to historical or other examples, such as Nazi-Germany for instance.<br>(*) "Organisations, governmental bodies or individuals may create and spread MDM for monetary incentives, ideological reasons, or to obtain short-run profit from attracting online attention (Townsend, 2016; Allcott & Gentzkow, 2017). The most frequent topics spread seem to be those of health, politics, and disaster (Muhammed & Mathew, 2022) and this may cause confusion and mislead people about important decisions about their lives and society (AdjinTettey, 2022)." (p. 2)			disinformation can be understood in the same way (i.e., through social cognitive processes, listing different theories and noting historical examples, as advised).<br>● We explain that disinformation and malinformation can both be understood as means of persuasion, and we also list various cognitive theories here.<br>● We also connect the terms to established constructs (e.g., disinformation and propaganda).<br>● We removed all references to historical examples, in line with Reviewer 2's suggestion, as their exclusion aligns the protocol more closely with contemporary interpretations and examples, and historical cases offer limited interpretative value in this context	
13. Description of the condition	The introductory sections on the problems of MDM and of MDM interventions could be improved in terms of scholarship. First, some critical claims (links between social media and MDM, links between authoritarian regimes and MDM, etc.) are treated only in passing with no citations of peer-reviewed publications and little interpretive value. Second, the MDM field has exploded in recent years – real-world examples, considerations about the role of social media, research methods, theories (beyond inoculation, which is given	R2	×	Thank you. We have addressed this feedback in several ways.<br>● We have re-drafted Section 1 (Background) and removed sentences on authoritarian regimes and social media, focusing instead on the relationship between MDM and the audience before introducing manipulation in a more general way.<br>● In Section 1 (Background) and Section 1.1, we include some real-world examples of	☐

	disproportionate focus), and (critically) methodological advances and debates (e.g., critical issues like backfire effects on accuracy perceptions of true information are addressed only in passing; are there any such methodological concerns in relation to VE outcomes?). Most of the cited studies in the introductory sections are relatively old by the standards of the field, which is not inherently a problem in reviews (more recent work is not necessarily more high-quality nor relevant), but it is in a field like this and on a topic that is evolving so rapidly. There are countless high-impact theoretical and empirical articles on MDM and experimental interventions in recent years. The background sections would benefit from a deeper and more thoughtful engagement with the empirical literature on MDM, interventions therein, active debates about the efficacy of different types of interventions, and – as mentioned above – the relation of this field to VE.			MDM in the context of radicalisation.<br>● We now speak in more detail about the role played by social media in Section 1 (Background) where we speak about channels, novelty, emotions and vulnerable audiences.<br>● In terms of the processing of incorrect or inaccurate information, we now draw from a wider arsenal of socio-cognitive theories in Section 1 (Background). We also draw from persuasion theories in this section to inform malintent.<br>● We have also re-drafted Section 1.1 and included, as advised, more recent empirical studies on misinformation and, notably, the role of misinformation in the process of radicalisation.	
14. Description of the condition Description of the condition	A critical limitation of this part is its failure to thoroughly establish a causal link between misinformation and violent extremism (i.e., "the problem") based on existing research. While both phenomena are discussed, their causal relationship receives too little attention. To my knowledge, the evidence base demonstrating direct causation remains sparse (let alone for intervention effectiveness, which further narrows down the literature). This gap may set a difficult premise for a comprehensive review as is planned here. Honestly, I am unsure about how much research the authors will be able to identify given the quite specific focus and early stage of the field.	R2	×	Thank you. We have now drafted Section 1.1 which better elucidates the relationship between MDM and violent extremism.<br>● Here, we argue that the relationship between the proliferation of false or misleading information and radicalization is somewhat established in that the former can play a critical role in extremist narratives. Here, we cite both empirical and theoretical studies to support this point.<br>● We also argue that those most susceptible to misinformation and, in particular, conspiracy theories, are those who already present with psychological vulnerabilities or	☐

				risk factors for radicalization. Here, we cite empirical literature from radicalisation and communication.<br>● We also connect resonance with MDM to the network elements of radicalisation, including the creating of "white masculinist paranoia".<br>● Finally, we speak about the behavioural outcomes which we have seen with those who have been exposed to MDM.	
15. Description of the condition	The framework for mis-, dis-, and malinformation would benefit from explicitly addressing conspiracy theories' classification and relationship to extremist violence. While the current taxonomy effectively distinguishes between the three forms, it overlooks conspiracy beliefs - a documented predictor of violent extremism supported by some empirical research and many case studies. Integrating conspiracy theories into this framework would strengthen its applicability to extremism research and better reflect real-world radicalization pathways.	R2	×	We have addressed this comment in two ways.<br>● In Section 1 (Background) we have specifically differentiated between the three, central concepts on theoretical grounds.<br>● In terms of a framework to explicitly address conspiracy theories we<br>● now draw from the intent/truth framework in Section 1 (Background) to suggest, in Section 1.1, that conspiracy theories could be classified as mis, dis, or malinformation depending on the intent of the communicator ("However, even if the information is "not false by definition", it may be "unverified, implausible and epistemically unsound" (e.g., conspiracy theories, see Brotherton & French, 2015, p. 1) and, depending on the intent of the communicator, could be classified as either misinformation, disinformation or malinformation").<br>● In relation to conspiracy theories as a	☐

				predictor of violent extremism, we have also completely re-drafted Section 1.1 which details the role of MDM in radicalisation. In capturing this literature, we have included several insights on conspiracy theories and radicalisation, including overlapping vulnerabilities in target audiences.	
16. Description of the condition	The term "information integrity" is suddenly introduced and becomes a key factor in the authors' model but it needs to be defined.	R2	×	Thank you. The initial title of this review included information integrity, but later we changed the title but did not change the text. Now all references to information integrity were removed to make the protocol clearer.	☐
17. General comments about the introductory sections	**Unclear Impact**: The introduction claims that the "speed, scale, and quantity of incorrect information has been associated with an unprecedented impact on society." I believe there is more evidence now that the actual societal impact remains unclear. Increasingly, it seems to be the continuous concern and discourse about misinformation that has had an impact, rather than the misinformation itself. The manuscript should reflect this growing body of evidence.	R3	×	Thank you. In Section 1 (Background) we make reference to the impact of MDM on society at different levels.	☐
18. General comments about the introductory sections	**Definitions**: The claim that terms like misinformation, disinformation, and malinformation are used interchangeably is no longer accurate. Most research begins with clear definitions and specifies what is and is not covered. For example, the distinction between misinformation (without intent) and disinformation (with intent) is standard. The authors cite Ireton and Posetti, but the first significant distinction was made by Wardle and Derakhshan (2017) in their "Information Disorder" report for the Council of Europe, which the authors should reference.	R3	×	Thank you. We appreciate this comment and have addressed it in the following ways.<br>● First, we no longer claim that the terms are used interchangeably.<br>● Second, we have included the Wardle & Derakhshan definition as advised.<br>● Finally, we have expanded upon the differences between the terms in Section 1 (Background), using both conceptual articles and, notably, theoretical lenses to do this.	☐
19. Section 1.1	**Weak Justification**: The authors argue that the link between MDM and violent extremism is under-	R3	×	Thank you. We have addressed this comment in two ways:	☐

	researched, but this is a weak argument. They should lead with real-world examples where misinformation has been linked to violence, such as the U.S. Capitol riots, rather than speculating or discussing risks without concrete evidence.			● First, we have expanded the theoretical and empirical premise of the link between MDM and violent extremism in Section 1.1.<br>● Second, we have included a real world example (i.e., "pizzagate") of MDM leading to violent extremist behaviour in Section 1 (Background)	
20. Section 1.1	**Definitions**: The definition of radicalization provided—"the process of developing extremist ideologies and beliefs — needs to be distinguished from action pathways — the process of engaging in terrorism or violent extremist actions"—is not a proper grammatical sentence and does not define radicalization. Instead, it vaguely notes what radicalization is not. The authors also fail to explain what they mean by "violent extremism" and "radicalization" early on, which is problematic. For instance, they start by discussing individual violent extremism on social media and then jump to "violent extremist organizations" in the last sentence. This shift is confusing and highlights how the manuscript frequently lacks focus and coherence.	R3	×	Many thanks for your helpful comments. We have re-drafted Section 1.1 to better introduce our key concepts.<br>● Section 1.1, we got rid of the detailed description of the two pyramids model and defined "radicalisation" in a more coherent way.<br>● To do this, we have also defined "extremism".<br>● We have also provided a more informed synopsis (drawing from the wider radicalisation and communication literature) on the degree to which MDM can reinforce (or "spark") violent extremism.	☐
21. Section 1.1	**Focus on MDM**: The authors do briefly discuss individual susceptibility to MDM, which I appreciate, but they should reflect on whether misinformation is truly the trigger or whether any type of provocative information—true or false—could lead to such behavior. What specifically about MDM amplifies these reactions?	R3	×	Thank you. We have included a discussion on what amplifies violent behaviour and violent extremism.	☐
22. Section 1.2	**Unclear Scope**: This section seems to focus on governmental interventions to tackle MDM, but this is not made clear upfront. Moreover, the authors later discuss content moderation by Big Tech, which is not a governmental intervention. This mix	R3	×	Thank you. We restructured this section to clarify the role played by governments/international organisations and big tech, and also their interconnectedness when tackling MDM.	☐

	of topics is confusing, and the authors need to clarify what this section covers and why.				
23. Section 1.3	**Unsubstantiated Claims**: The authors claim that “the most used is the inoculation strategy,” but where is the evidence for this? Fact-checking is far more common, with nearly all major media organizations now employing dedicated fact-checking teams. The authors list several interventions, but these could be better categorized into reactive (e.g., fact-checking) and proactive (e.g., prebunking, media literacy) strategies, highlighting their respective purposes and trade-offs.	R3	×	Thank you. We corrected that sentence. We also divided the interventions in these two distinct categories and briefly discussed their purposes and trade-offs.	☐
24. Section 1.3	**Violent Extremism Link**: Violent extremism is reintroduced at the end of the paragraph, but the connection between interventions and violent extremism remains underexplored. The manuscript needs to address this explicitly.	R3	×	Thank you. This point has been addressed in several ways in Section 1.1.	☐
25. Section 1.3	**Figure 1**: Did the authors create this figure themselves? If so, it should be more informative. As it stands, it does not add much to the manuscript.	R3	×	Yes, it was created at the very beginning of this review, at the Title Registration From. And we agree, it does not add much to the protocol, so we decided to remove it.	☐
26. Section 1.4	**Mischaracterization**: The authors incorrectly describe prebunking as involving a weakened form of MDM. In reality, it usually takes the form of a generic warning. Prebunking and inoculation should not be conflated, as they have distinct mechanisms and effects. Additionally, recent studies have found that warnings may increase skepticism even toward accurate information, which should be acknowledged.	R3	×	Thank you. We corrected that information and included a discussion on adverse effects.	☐
27. Section 1.5	**Unclear Contribution**: The stated contribution—to examine which interventions are most effective against violent extremism—should be introduced much earlier. However, this paragraph also introduces “information integrity,” which was not mentioned previously. This inconsistency adds to	R3	×	Thank you. The initial title of this review included information integrity, but later we changed the title but did not change the text. Now all references to information integrity were removed to make the protocol clearer.	☐

	the overall lack of focus in the manuscript.				
28. Description of the intervention	The intervention is described clearly, making it easy for anyone unfamiliar with it to understand. However, to make it more comprehensive and impactful, **some other approaches worth exploring further.**<br>● **Role of social media Platforms**: Emphasize the accountability of social media platforms in developing and enforcing policies minimize MDM spread.<br>● **Real-time Monitoring Tools**: Highlight the use of advanced analytics and real-time monitoring systems for identifying and countering MDM as it spreads.<br>● **Interventions reflected in other systematic reviews**: Incorporate findings from other systematic reviews, such as IPIE's "Strategies for Improving the Global Information Environment: Results from a Systematic Review and Meta-Analysis" (2023) and Carnegie "Countering Disinformation Effectively: An Evidence Based Policy Guide (2024), These studies discuss interventions like labelling social media content and counter-messaging strategies distinct from inoculation and fact-checking, which are not currently mentioned.	R1	×	Thank you. We followed your suggestion and included the mentioned strategies and a discussion on the role of social media platforms.	☐
29. Description of the intervention	On **page. 8**, the text outlines primary, secondary, and tertiary prevention strategies for VE. To improve this section, consider adding clarifications:<br>● **Primary Prevention:** Focuses on community-wide strategies to build resilience to VE. It also supports narratives that counter extremist ideologies.<br>● **Secondary Prevention:** Aims to prevent vulnerable individuals from engaging in violent extremism.<br>● **Tertiary Prevention:** Involves developing strategies to disengage or deradicalize those individuals already engaged in violent extremism and may include rehabilitation efforts.<br>● **Suggestions:** Clarify how interventions addressing	R1	×	Thank you. Following your suggestion, we clarified these three distinct prevention strategies.	☐

	MDM fit within these prevention levels. While they mostly align with primary prevention, they could also be applicable in secondary prevention.				
30. Description of the intervention	The review primarily focuses on MDM in relation to VE, but the theoretical connection between these constructs is underspecified. Key points to address:<br>● It is not clear how MDM directly influences VE, as much as VE (attitudinal or behavioural) may not stem from exposure to manipulative or false information.<br>● The current discussion of this connection is minimal. For example, Figure 1, intended to illustrate the theoretical relationship, lacks sufficient theoretical grounding.<br>● **Recommendation:** Strengthen the theoretical framework by engaging with exciting literature and empirical studies on the relationship between MDM and VE. Clarify why MDM interventions are exposed to impact VE, especially when tested on the general population.	R1	×	Thank you. We have provided more clarification on this in Section 1.	☐
31. Description of the intervention	The review's distinction between inoculation and prebunking is inaccurate in the introduction (p. 7), as inoculation is a specific prebunking technique. This inconsistency should be resolved, aligning with the later correct usage where inoculation is presented as an example of prebunking.	R2	×	Thank you. We have corrected those different strategies.	☐
32. Description of the intervention	Also, it is surprising that there are no supporting references for statements such as "The most used is the inoculation strategy, but others, such as pre-bunking and debunking, fact-checking, media literacy, gamification, and educational and informational interventions are also used to tackle the effects of MDM." Without references to previous reviews of the field, this remains subjective.	R2	×	Thank you. You are right. We corrected this sentence and added some references.	☐
33. Description of	It is also unclear what is meant with "ultimately to promote the ability to stop the dissemination of	R2	×	Thank you. We explained a bit further.	☐

the intervention	MDM". Whose ability? Policymakers? Users?				
34. Description of the intervention	Why would interventions only be implemented with individuals aged 18 years and above? As long as ethical approval was obtained, shouldn't studies with younger populations also be considered?	R2	×	Thank you. We remove the age barrier as some studies might indeed include minors.	☐
35. Description of the intervention	This also remains speculative without references: "The interventions used against MDM usually fall under the first level, but can also be found in the second level."	R2	×	Thank you. We removed that sentence.	☐
36. Description of the intervention	The background would benefit more generally from a clearer taxonomy of intervention strategies and what their proposed mechanisms are. We should have early exposure to a working definition of what relevant interventions are in this section. This is even more important when we are introduced to 'types of interventions' in the methods section, which relies primarily on examples rather than a precise definition.	ME	×	Thank you. The definitions of the different strategies were provided in Section 1.4 to avoid unnecessary repetition throughout the paper.	☐
37. How the intervention might work	The description of how the intervention might work is present but lacks depth. It is argued that certain interventions may prevent individuals from succumbing to disinformation that could lead to radicalization. However, the connection between belief in disinformation and the process of radicalization remains unclear. For instance, even if a strategy successfully prevents some individuals from believing in disinformation, it is uncertain whether this group overlaps with those at risk of radicalization. These groups may be entirely distinct. The systematic review focuses on studies measuring the effects of interventions on violent extremism (VE) but does not provide a logic model to clarify how anti-disinformation measures	R1	×	Thank you. Additional information was added to provide further insight into the mechanisms behind the intervention's influence on MDM in extremism prevention.	☐

	influence VE.				
38. How the intervention might work	Additionally, there is no discussion of the continuum of VE or how different interventions might vary in their impact across this spectrum.	R1	×	Thank you. We added some paragraphs discussing the continuum on VE and how the timing of interventions might impact across this spectrum.	☐
39. How the intervention might work	On page 9, the authors write that "…individuals are exposed to a mild version of a false claim to strengthen their defences against future misinformation." It is worth noting that these types of 'prebunking' techniques are also often paired with an educational campaign and media literacy tips to educate the public on how to spot MDM. This is worth clarifying as the exposure must also be done alongside education in order to not further the spread of MDM narratives.	R1	×	A statement was added on refutational preemption with the aim of clarifying the additional information that can be given in this type of intervention.	☐
40. How the intervention might work	The distinction between three major categories of interventions (i.e., prebunking, debunking, and media literacy) needs to be anchored in research. There are large scale reviews of the different types of interventions against misinformation, which usually include further categories. See, e.g.,<br><br>https://doi.org/10.1038/s41562-024-01881-0<br>https://doi.org/10.1145/3674724<br>https://doi.org/10.1027/1864-1105/a000407	R2	×	Thank you. We have provided definitions and references for these strategies.	☐
41. How the intervention might work	The review neglects a critical concern: potential adverse effects of anti-misinformation interventions on general information processing. Research indicates that inoculation and similar approaches sometimes may inadvertently foster broad skepticism, leading people to reject legitimate information and possibly in the long run erode trust in democratic institutions. This risk warrants systematic evaluation through empirical measures that assess both intervention effectiveness and	R2	×	Thank you. The adverse effects were referred to in a new sentence.	☐

	potential negative impacts on information acceptance and institutional trust.				
42. How the intervention might work	You write about debunking, "This type of intervention can be time-intensive and may not completely counteract the effects of misinformation. In practice, debunking efforts do not always reach those who need them most, as individuals who are predisposed to believe falsehoods often avoid corrective information (Hoes et al., 2024)." However, similar problems (outreach, time intensity, small effects) apply to inoculation and should be noted (see https://doi.org/10.56296/aip00019). Generally, I am not aware of any interventions that "completely counteract" misinformation effects.	R2	×	The following sentence was added in order to clarify any doubts, "such as the other available interventions".	☐
43. How the intervention might work	The review lacks critical details about the processes the interventions build on. While a logic model is suggested, the review fails to outline specific intervention mechanisms, processes, and causal pathways. A clear theoretical framework showing how each intervention achieves its intended effects would strengthen the proposal.	R2	×	Thank you. This suggestion was included in response to previous comments.	☐
44. Why it is important to do the review	There is a general discussion of anti-disinformation interventions in general but it does not include some of the most significant meta-reviews of these interventions that have already been done, i.e. IPIE (2023) and Carnegie (2024) cited above. In general, the research tends to be older rather than newer information.	R1	×	Thank you for identifying these two reviews. We have included them in the protocol in this section.	☐
45. Why it is important to do the review	While it stands to reason that 'how to prevent VE' could be a gap in the disinformation domain, the relationship between disinformation and VE, and the transferability of interventions from the former to the latter, is not characterized.	R1	×	Thank you. We added three sentences where we explain how the interventions on MDM can hold promise to prevent VE.	☐
46. Why it is	Very little primary research is cited in the importance section, which links back to my concern	R2	×	Thank you. Per protocol definition, this section only includes review studies. For that reason, following	☐

important to do the review	that there really may not be much research – not even work showing a causal link between misinformation and violent extremism, leave alone testing interventions. Some additional reviews of relevance should also be considered, e.g., https://doi.org/10.1177/14407833241231756<br><br>**Editorial comment:** Primary studies should only be listed here if they show disagreement/inconsistencies, gaps, or other important issues that need to be settled by a review. Otherwise, your focus needs to be on previous reviews and police/practice importance of the review.			the Editorial comment, we will not include primary studies in this section.	

**Objectives Only**	**Feedback**	**Reviewer**	**Revised?**	**Author comments**	**ED Use**
48	The objectives for the review of guiding practice and policy are indicated but confidence is not provided that the outcomes of the review will be useful in these regards.	R1	x	We have refined the objective provided the feedback and a discussion with the funders.	☐
49	The topic of investigation is clearly of relevance to policymakers, practitioners, and societies at large. This is well articulated. Minor point: In the Objectives section, a "reduced consensus" is used to justify the review. However, without any reference, this remains very vague and subjective.	R2	x	We have refined the objective provided the feedback and a discussion with the funders.	☐
50	The research questions do not flow logically from the preceding text. Up to this point, the manuscript reads as fragmented and disorganized. A significant revision is needed to ensure that the questions are clearly motivated by the text.	R3	x	We have refined the objective provided the feedback and a discussion with the funders.	☐

**Methods Only**	**Feedback**	**Reviewer**	**Revised?**	**Author comments**	**ED Use**
51. Criteria for considering	In this review, you have chosen the typology where misinformation is defined as unintentional false	R1	x	Thank you for highlighting the issue of typological variation in the definitions. To address this, we have	☐

studies for this review	information, disinformation as intentionally false information, and malinformation as accurate information shared out of context to harm, manipulate, or confuse. However, in other papers, different typologies may be used, or the may not clearly explain it. For example, in many works, the term "misinformation" is used broadly and encompasses all types of false information. The issue arises because there is no universally accepted or consistent typology for these terms. While this is largely true for older papers or those published a few years ago, it is less common in newer studies, which tend to adopt more consistent typologies. Our concern is: how do you ensure that all selected papers align with the same typology? If not, how do you plan to address the variations in definitions across the papers included in your review.			adopted explicit definitions for each term in our background section, and these definitions consistently underpin our analysis throughout the review. Recognizing that the literature does not use universally accepted definitions, especially in older studies, we intend to carefully documented the operational definitions used in each included study wherever they differ from our specified typology. Where necessary, papers that use broader or alternative uses of "misinformation" will be flagged and discussed in relation to our framework, and their findings will be contextualized accordingly. Furthermore, by providing clear definitions of radicalization and extremism in Section 1.1, we clarified inclusion and exclusion criteria.	
52. Criteria for considering studies for this review	The criteria make sense if "quasi-experimental designs" include field studies of the real-world effectiveness of interventions against MDM--which in my understanding tend to have more authority in this domain than lab-based studies. I was not totally clear from this language if field-based observational studies were meant to be included.	R1	×	Thank you for the opportunity to clarify this point. We have chosen to include only experimental and quasi-experimental studies in this review based on concerns related to exposure control. Specifically, in the context of MDM, exposure is highly variable and difficult to control in real-world settings. As such, the absence of an observed intervention effect in an observational study may not necessarily indicate that the intervention is ineffective, but rather that the exposure to MDM differed substantially across participants in ways that cannot be accounted for.<br>To clarify: quasi-experimental designs may include field-based studies, provided they involve some form of intervention manipulation and/or a comparison group that allows for causal inference (which is adequately addressed in the manuscript). Our intention is not to privilege lab studies, but to prioritise study designs, whether in the lab or the field.	☐

				However, we view this review as an initial step in mapping the existing evidence on the effectiveness of interventions. Establishing which interventions show promise under more controlled conditions will help inform subsequent translational work, including observational evaluations of real-world implementation.	
53. Criteria for considering studies for this review	A further discussion on why prevention is hard to measure could be useful here. It also provides justification for the need for a mixed methods approach.	R1	x	Thank you. We included a brief paragraph to discuss why it is difficult to measure prevention, but we did not discuss the need for a mixed methods approach, because, as agreed with our funders, we will leave out qualitative studies.	☐
54. Criteria for considering studies for this review	The focus on MDM in the context of violent extremism is a relatively recent research topic, with many interventions being implemented maybe only in the past few years. This may result in a lack of published studies or data, especially those that use robust methodologies and have undergone peer review. Revisit the exclusion of qualitative research or include a plan to integrate findings from qualitative studies in a mixed-methods approach. This would provide a fuller picture of the context and mechanisms through which interventions operate.	R1	×	Thank you for this comment. We fully agree that qualitative research can offer critical insights into the contextual and mechanistic dimensions of interventions targeting misinformation (MDM) in the context of violent extremism. However, the current review has been built as a focused synthesis of the existing quantitative evidence, specifically limited to experimental and quasi-experimental studies. This decision was made to identify interventions that show preliminary causal effects, thereby laying the groundwork for future, more integrative reviews. As such, while we recognise the value of including qualitative evidence or adopting a mixed-methods approach, these elements fall outside the remit of the current project.	☐
55. Criteria for considering studies for this review	The article selection process makes no attempt to focus on vulnerable populations, though perhaps some identified studies will include such participant samples. Are there any/enough studies testing MDM interventions on VE-vulnerable populations with VE-related outcomes? If not, should this review explicitly state up front that it is about prevention in the overall population, and avoid setting up an expectation of evidence for countering or deradicalizing? In any case, it is worth	R1	×	Thank you. At this point we cannot say for sure if there are enough studies testing MDM interventions on vulnerable populations. However, if we do find, we will acknowledge that this review is about general population and that our results do not apply to vulnerable populations. But we can only confirm once the analysis is finished. However, as can be seen in Section 3.1.2, there are no restrictions regarding the target population.	☐

	acknowledging that meta-analytic findings surfaced here may not apply to individuals most likely to commit VE.				
56. Criteria for considering studies for this review	One major aspect requires clarification. It is unclear whether the focus of the review is on: 1) interventions' effects on violent extremist attitudes/behaviors, or 2) belief reduction in extremist misinformation. The inclusion of studies like Lewandowsky & Yesilada (2021) suggests both outcomes are considered, introducing potential methodological concerns about common method variance and response bias that should be addressed. To me, 2) seems better suited as a manipulation check than an endpoint, but the authors may have well-justified reasons for including it.	R2	x	We confirm that the primary focus of the review is on the effects of interventions on violent extremist attitudes and behaviours. The additional clarity provided in the revised outcome measures section and the reframed background aim to address this issue directly. We hope these changes resolve any ambiguity regarding the review's scope.	☐
57. Criteria for considering studies for this review	We need considerably greater detail on relevant outcomes, even more than was provided after the last action letter. What is an attitudinal measure? What is a behavioural measure? You provide some information already but a clearer and more structured discussion is essential to this review's credibility.	ME	×	Thank you for your feedback. In response, the revised manuscript now provides clear definitions of attitudinal and behavioural outcome measures, drawing directly from the radicalisation literature.	☐
58. Data collection and analysis: Search	The search terms seemed too narrow. Searching for interventions such as factchecking, literacy training etc. seem to situate the estimate, especially insofar as other interventions against MDM are not included in these search terms, such as content labelling and other nudge techniques, as well as a wider scope of counter-messaging and corrective information strategies described in the IPIE and Carnegie Report cited above.	R1	×	Thank you for your comment. While we recognise that the suggested search terms could be relevant, our search strategy already encompasses a wide range of interventions that have been validated by the editorial team. In addition, we have incorporated multiple supplementary mechanisms to identify further studies, including both backwards and forward reference harvesting.	☐
59. Data collection and analysis: Search	The terminologies listed_ disinformation, fake news, mailinformation, misinformation, mistrust, and propaganda are a good starting point for the systematic review. However, to ensure a more comprehensive and nuanced analysis, we recommend expanding the scope to include	R1	×	Thank you for your comment. While we recognise that the suggested search terms could be relevant, our search strategy already encompasses a wide range of interventions that have been validated by the editorial team. In addition, we have incorporated multiple supplementary mechanisms	☐

	additional terms such as **rumor, fabricated information, and information literacy**, etc. These terms capture other dimension of information distortion and could provide valuable insight when reviewing literature. For a more comprehensive discussion, you may refer to "A literature review on detecting, verifying, and mitigating online misinformation. (2023)", **Figure 1.** which provides detailed insights into various types of misinformation, disinformation, and fake news.			to identify further studies, including both backwards and forward reference harvesting.	
60. Data collection and analysis: Search	I also am not convinced about the distinction between Categories 2 and 3. Let's say a study is a hit according to Category 1 and Category 2 (i.e., about "misinformation" and "inoculation", respectively) but then for some reason fails to mention terms in Category 3. In such a case a study "testing the effect of an inoculation procedure on misinformation beliefs" would be excluded, which seems suboptimal.	R2	×	Thank you for your observation. We would like to clarify that Category 3 terminology, related to study design, is not limited to the title, but is searched across multiple fields, including the abstract, author-supplied keywords, and subject/indexing terms. This approach is grounded in principles of scientific reporting, wherein abstracts of experimental or quasi-experimental studies are expected to include some reference to key elements of study design, such as group allocation, intervention, control, or randomisation. The terms included in Category 3 were developed to capture this range of expressions, ensuring that studies employing such designs are identified even if not explicitly labelled as "RCT" or "experiment" in the title. While it is possible that a study meeting Categories 1 and 2 could omit all relevant design terms in the abstract or indexing fields, we consider this unlikely given reporting standards in the literature.	☐
61. Data collection and analysis: Search	It could help to specify the language inclusion criteria.	R2	×	This has now been added to the review, as no exclusions were made based on language.	☐
62. Data collection and analysis:	The additional detail on selection of studies is welcome. However, you are advised to avoid excluding studies based on outcome at title/abstract	ME	×	Thank you for your note. As we conducted the title and abstract screening for this review concurrently with the one by Duarte, we adhered to your	☐

Selection of studies	stage as it is rare that all outcomes reported in a study are reflected in the abstract.<br><br>**Editorial comment:** The Methods Editor is correct and in our haste to progress your protocol and consider a dual screening approach for your and Duarte's review, we did not consider this issue. As has been advised to the Duarte team, we require the combined Title/Abstract screening process to be as follows. You should have only three options to choose from when screening:<br>1) This record could relate to an education program for reducing extremism/radicalization (goes to Duarte review full-text screening)<br>2) This record could relate to addressing MDM (goes to de Carvahlo full text screening)<br>3) This record does not relate to either of the topics in 1) or 2).<br><br>Once you have completed the screening of titles and abstracts (preferably through Eppi's priority screening functionality), each set of records relating to 1) and 2) can then be imported into separate reviews to proceed to screening on full texts. EppiReviewer developers can help you to set this up. Please edit this section to reflect this process.			recommendation and did not exclude studies based on outcomes during the initial screening phase. This has been added to the protocol.	
63. Data collection and analysis: Selection of studies	The article search process says very little about how the massive field of MDM will be boiled down to studies that specifically have VE-related outcome variables. It sounds like the authors will conduct a fairly comprehensive search of all MDM intervention experiments, and then later manually identify those with VE-related outcomes. Presumably these VE-focused MDM studies make up a tiny fraction of all MDM studies. Only a few examples of VE-related outcome measures are reported, and some (e.g., Exposure to Violent	R1	×	Thank you. Following the description made for the Duarte's review, we have also provided similar detail, adapted to this review.	☐

	Extremism Scale) sound like they are not amenable to MDM interventions anyway (i.e. how would an in-the-moment MDM intervention like prebunking shape exposure?<br><br>**Editorial comment:** Additional detail is required for how screening will be conducted – in an operationalised way – at full-text (see also my comment on the selection process re: intersection with Duarte's review).				
64. Data collection and analysis: Assessment of risk of bias	"No studies will be excluded based on results from the risk of bias assessment, though considerations will be made in the findings, to take into account higher levels of bias and their potential impact on the results of the review." This is rather unclear. It should be specifically explained how the influence of bias will be formally assessed. Later in the sensitivity section, it becomes clear that tests will be conducted. It would therefore be good to streamline this part for consistency.	R2	×	Thank you. To improve clarity and consistency, we have revised the final paragraph of the Risk of Bias section to explicitly state that the potential influence of bias will be formally assessed through the planned sensitivity analyses described later in the protocol.	☐
65. Data collection and analysis: Unit of analysis issues	It is not clear how an ICC will be calculated/obtained from interventional studies that usually consist of two groups.	R2	×	Thank you. We sought to clarify this by explicitly stating that this unit of analysis issue only arises when allocation or delivery occurs at the cluster level, therefore excluding two individually randomised groups (without clustering).	☐
66. Data collection and analysis: Unit of analysis issues	It appears that in responding to the prior action letter, important information relating to how outcomes will be grouped by follow-up time has been lost. This should be inserted in the 'Unit of analysis issues' section, clarifying specifically how you will group timepoints for analysis.<br><br>**Editorial comment:** To be clear, content relating to time-points belongs in this section. Content related to multiple conceptually similar outcomes for a study (not related to time-point) should be in the 'Criteria for determination of independent findings'	ME	x	We apologize as we seemed to misunderstood previous action letters. All content relating specifically to time-points has been included exclusively in the revised 'Unit of analysis issues' section, while the 'Criteria for determination of independent findings' section now solely addresses approaches for multiple conceptually similar outcomes at the same timepoint.	☐

	(e.g., two effect sizes at post-intervention for depression). Please ensure this section and section 3.3.7 are correctly written in this way.				
67. Data collection and analysis: Unit of analysis issues	There is no need to apply the ICC again if an effect size in a study already contains a 'cluster-adjusted' effect estimate.	ME	×	This has been clarified. Thank you.	☐
68. Data collection and analysis: Criteria for determination of independent findings	While I note you will use Lopez-Lopez (2018) as a framework for handling dependencies, I would strongly suggest specifying now that you will use robsut variance estimation meta-analysis to handle situations where studies contribute multiple relevant effect sizes to an analysis. This method has come to prominence as the optimal choice since the publication of Lopez-Lopez et al (2018), particularly given the 'integrative' focus of your review. Of note as well is that robust variance estimation precludes needing to merge intervention or control groups if multiarm trials are presented; each relevant pairwise of an intervention vs control can be entered into the analysis (though I would still recommend merging control groups).	ME	x	Thank you for your suggestion. The protocol has been revised to specify that robust variance estimation meta-analysis will be implemented to address statistical dependencies arising when studies contribute multiple relevant effect sizes. We also added the information on robumeta.	☐
69. Data collection and analysis: Unit of analysis issues	There is no need to apply the ICC again if an effect size in a study already contains a 'cluster-adjusted' effect estimate.	ME	×	This comment is the same as 67 and has thus been addressed.	☐
70. Data collection and analysis: Data synthesis	The authors state "We will use the dmetar package from R. / The meta-analyses will be performed using the dmetar package for R." However, this seems not to be a standalone package but an addon to more common packages such as those handling three level analysis (e.g., metafor) or robust estimation (e.g., robumeta).	R2	x	We acknowledge that the dmetar package is not a standalone tool, but a companion resource built on the core statistical infrastructure of packages such as metafor and robumeta. We have revised the text throughout the manuscript to clarify that all primary meta-analytical and heterogeneity analyses will be conducted using metafor (and robumeta where appropriate), with dmetar used only for supplementary functions and graphical outputs.	☐

Key to Reviewers = ED: Editor; AE: Associate Editor; ME: Methods Editor; IR: Information Retrieval specialist; R1: Reviewer 1; R2: Reviewer 2.

71. Data collection and analysis: Data synthesis	I strongly recommend reporting 90% Prediction Intervals (in addition to 95% CIs) to gauge the consistency of effects.	R2	×	Thank you for the suggestion. This has now been added to the protocol.	☐
72. Data collection and analysis: Data synthesis	"For outcomes that cannot be included in the meta-analysis due to incompatible conceptual frameworks or significant heterogeneity, we will instead present standardised individual effect sizes." I am not sure I understand this. Why would studies be excluded based on "heterogeneity"?<br><br>**Editorial comment:** It will be important to be clear on what you mean by this. Do you mean outliers will be excluded from meta-analysis or do you mean that conceptually unique studies that cannot be operationally grouped with other studies (e.g., perhaps because of population or other intervention characteristics).	R2	×	Thank you for flagging this ambiguity. Our intention was not to exclude studies because of statistical heterogeneity. We have revised the text to state that studies will be withheld from pooling only when they are conceptually unique, for which we will present standardized individual effect sizes and a structured narrative synthesis.	☐
73. Data collection and analysis: Data synthesis	A critical moderator to be considered is the length of follow-up. For instance, inoculation effects are known to fade quickly without boosters.	R2	x	This dimension was explicitly included in the corresponding section.	☐
74. Data collection and analysis: Data synthesis	As advised in the previous action letter, you are strongly recommended to avoid undertaking 'stratified' meta-analyses by study characteristics. This introduces unnecessary instability into outcome estimates because it reduces the information available for estimation of between-study variance parameters (tau-squared).	ME	x	Thank you for your recommendation. We have revised the methods to clarify that moderator effects will be examined exclusively using meta-regression.	☐

Other comments Only	Feedback	Reviewer	Revised?	Author comments	ED Use
75. Any other general comments	In its current form, the manuscript requires substantial revision to:<br>1. Justify the need for a systematic review clearly and persuasively.	R3	×	Thank you for your review. We have made substantial revisions to the manuscript in response to your feedback and that of your peers.	☐

	2. Provide a coherent structure with logical progression and clear connections between topics.<br>3. Address critical omissions, such as the scope of MDM, the role of intent in interventions, and the effectiveness of measures online versus offline, which is particularly relevant for governmental strategies<br>4. Avoid unsubstantiated claims and alarmist tones.<br>I encourage the authors to reorganize their arguments, strengthen their focus with real-world examples, and clarify the purpose and contribution of the manuscript.				
Unit of Analysis Issues	Regarding the following: “Following this appraisal, we will combine the groups to form a single pairwise comparison, adhering to the recommended approaches outlined by Higgins et al. (2019) for both dichotomous and continuous outcomes”. I will await methods review on this, but it is not always appropriate to combine treatments into one arm, especially if they are conceptually different interventions. While I appreciate that the Cochrane Handbook recommends this, it recommends this based on a very different example than what we have here. For example, combining an intervention arm that delivers an intervention online with an intervention arm that delivers an intervention in person would lose critical information about these two different intervention approaches. However, combining two eligible control groups might be relevant. As the Cochrane Handbook notes, an issue with this overall approach is that you can introduce heterogeneity by combining different intervention	Elizabet Eggins	×	In response, the protocol now specifies that intervention arms will only be combined for meta-analysis when they are sufficiently comparable in content and delivery. When substantial differences exist they will be kept distinct in the analysis to avoid loss of critical information and undue heterogeneity.	

	models into one arm. We will leave this one for the action letter after peer review and provide guidance on the best approach.				
