# Supplementary material for: PROTOCOL: Interventions Targeting Misinformation, Disinformation and Malinformation for Reducing and Countering Violent Extremism: A Systematic Review
Source: Campbell Syst Rev. 2026 Jun 21;22(2):18911803261459099. doi: 10.1177/18911803261459099 (PMC13305521; doi:10.1177/18911803261459099)
Supplement: Supplemental Material - Interventions Targeting Misinformation, Disinformation and Malinformation for Reducing and Countering Violent Extremism: A Systematic Review [file sj-pdf-2-cam-10.1177_18911803261459099.pdf]

## Appendices

### Appendix 1. Example Search Strategy

Database: APA PsycInfo <1806 to November 2024 Week 2>

Search Strategy

| # | Searches |
|---|---|
| 1 | (disinformation* or "fake news" or malinformation* or misinformation* or misperception* or mistruth* or propaganda*).ti,ab,id. (9895) |
| 2 | (corrected or correcting or correction* or counter* or debunk* or deplatform* or de-platform* or detect* or disprov* or educat* or "fact check" or "fact checking" or "fact checked" or "fact checks" or "fact-check" or "fact-checking" or "fact-checked" or "fact-checks" or gamif* or inoculat* or "literacy training" or "media literacy" or persuad* or persuas* or prebunk* or prevent*).ti,ab,id. (1219984) |
| 3 | (randomi* or randomly or rct* or allocat* or assign* or "between subjects" or blind or blinded or "control group" or "controlled study" or experiment* or "factorial design" or group or groups or interven* or matched or nonrandom* or non-random* or "non random" or "non randomised" or "non randomized" or "non randomly" or "open label" or open-label or placebo* or "pre-post" or quasiexperiment* or quasi-experiment* or quasirandom* or quasi-random* or trial* or "within |

| | |
|---|---|
| | subjects").ti,ab,id. (2072794) |
| 4 | and/1-3 (1727) |

**Appendix 2. Full-text Coding Form**

Eligible studies that are object of full text review will be first organized alphabetically, thereafter coded with a unique alphanumerical code preceded by a # (e.g, #01, #02, …). This code will be the Report ID for the purposes of extraction.

I.General Information

| | |
|---|---|
| a. Study ID<br><br>(surname of first author + year of publication) | |
| b. Report ID | |
| c. ID of Person Extracting Data | |

| | |
|---|---|
| d. Date form completed<br><br>(dd/mm/yyyy) | |
| e. Reference Identifier<br><br>(e.g., DOI, URL, …) | |
| f. Publication Type<br><br>(e.g., peer-reviewed journal article, dissertation/thesis, conference proceeding, book chapter…) | |
| h. Funding? | Yes<br><br>No<br><br>No information |
| h.1. Funding details<br><br>(if applicable) | |
| Notes: | |

II.Study Details

| a. Study Characteristics | | Location in text or source<br>(page and paragraph/fig/table/etc.) |
|---|---|---|
| a.1. Study Design | Experimental<br>Quasi-experimental | |
| a.2. Factorial Design<br>(e.g., number of intervention groups x number of control groups) | | |
| Notes: | | |

| b. Participants | | Location in text or source<br>(page and paragraph/fig/table/etc.) |
|---|---|---|
| b.1. Description | | |
| b.2. Recruitment Method | | |

| | | |
|---|---|---|
| b.3. Inclusion Criteria | | |
| b.4. Exclusion Criteria | | |
| b.5. Baseline imbalances | | |
| b.6. Withdrawals and exclusions | | |
| b.8. Sample Size Calculation | Yes<br>No | |

| b.7. Sample Size<br>(add “comparison” columns depending on the number of arms in the study) | | | |
|---|---|---|---|
| Number of participants | Intervention | Comparison | Total |
| Recruited | | | |
| Consented | | | |

| | | | |
|---|---|---|---|
| Began the intervention | | | |
| Completed the intervention | | | |
| Completed follow-up<br><br>(if applicable) | | | |
| b.8. Sample Features<br><br>(add "comparison" columns depending on the number of arms in the study) | | | |
| Number of participants | Intervention | Comparison | Total |
| Gender (% male) | | | |
| Age (M, SD) | | | |
| Ethnicity (if available) | | | |
| Religion (if available) | | | |

| | |
|---|---|
| | Location in text or source<br><br>(page and paragraph/fig/table/etc.) |

| | | |
|---|---|---|
| b.9. Baseline Imbalances | | |
| b.10. Withdrawals/Exclusions | | |
| Notes: | | |

| c. Intervention | | Location in text or source (page and paragraph/fig/table/etc.) |
|---|---|---|
| c.1. Allocation | Randomised<br>Matched<br>Score-Based<br>Other: ____________________ | |
| c.1.1. Additional information | | |

| | | |
|---|---|---|
| c.2. Unit of allocation<br><br>(for clusters: include the number of clusters) | Individual<br><br>Cluster (n = ___)<br><br>Other: ____________________ | |
| c.3. Setting | Community<br><br>University/School<br><br>Online<br><br>Other: ____________________ | |
| c.4. Designation<br><br>(include verbatim designation of the intervention group) | | |
| c.4.1. Theoretical Framework<br><br>(include key references) | | |

| | | |
|---|---|---|
| c.4.2. Description<br><br>(with sufficient detail for replication e.g., content, components, …) | | |
| c.4.3. Duration (in days, from recruitment to follow-up)<br><br>Obs. If intervention/comparison are conducted in a single moment please fill in with “0”. | | |
| c.4.4. Provider Characteristics<br><br>(include number, profession, training, and other relevant information) | | |

| | | |
|---|---|---|
| c.4.5. Co-interventions | No<br>Yes. Which?<br>________________<br>_______________________<br>Not enough information | |
| c.4.6. Delivery Mechanism<br>(means, intensity, fidelity) | | |
| c.4.7. Compensation for Participation | No<br>Yes. Which?<br>________________<br>_______________________<br>Not enough information | |

| | | |
|---|---|---|
| c.4.8. Cost of Intervention | No<br>Yes. Which?<br>________________<br>_______________________<br>Not enough information | |
| Notes: | | |

(repeat table from c.4. onwards according to the number of interventions and comparison groups defined in a.2.)

| d. Outcome | | Location in text or source (page and paragraph/fig/table/etc.) |
|---|---|---|
| d.1. Outcome | Violent Extremist Behaviour<br>Violent Extremist Attitudes<br>Both | |
| d.1.2. Additional information | | |

| | | |
|---|---|---|
| d.2. Time-points measured (select all that apply) | Pre<br>Post<br>Follow-up | |
| d.2.1. Number of follow-ups | | |
| d.2.2. Follow-up time-points | | |
| d.3. Measurement | Self-report<br>Interview<br>Other: ____________________ | |
| d.4. Measurement Tool | | |
| d.4.1. Validity | | |
| d.4.2. Reliability | | |
| d.4.3. Upper and lower | | |

| | | |
|---|---|---|
| limit | | |
| d.4.5. Thresholds | | |
| d.4.6. Interpretation | | |
| d.4.7. Missing data imputation | Listwise<br>Pairwise<br>Imputation by the mean/mode<br>Single regression<br>Dummy variable<br>No information<br>No missing data<br>Other: ________________________ | |
| d.4.8. Power achieved | | |
| d.4.9. Statistical Significance | Yes<br>No | |

| | | |
|---|---|---|
| d.4.10. Direction of the outcome | Positive<br>Negative<br>No change | |
| d.4.11. Group favoured<br>(refer to designations from c.4.) | | |
| d.4.11. Main conclusions | | |
| Notes: | | |

(when more than one outcome measured, repeat table from d.2. according to the number of outcomes.)

| e. Effect size | | Location in text or source<br>(page and paragraph/fig/table/etc.) |
|---|---|---|
| e.1. Total sample for the | | |

| | | |
|---|---|---|
| outcome | | |
| d.1.1. Total sample for treatment group | | |
| d.1.2. Total sample for control/comparator | | |
| (add lines corresponding to the number of control/comparator groups [d.1.3., d.1.4., …) | | |
| d.2. Attrition | Yes<br>No | |
| d.2.1. Attrition details | | |
| d.3. Raw effect size (value) | | |
| d.4. Standardized effect size | Yes<br>No | |
| d.4.1. Value | | |

| | | |
|---|---|---|
| d.5. Measure used | Mean<br><br>Standard Deviation<br><br>Standard Error<br><br>Proportion/Frequencies<br><br>Other:<br>________________ | |
| d.5.1. Mean value (treatment) | | |
| d.5.2. Mean value (control/comparison) | | |
| (add lines corresponding to the number of control/comparator groups [d.5.2., d.5.3., …) | | |
| Notes: | | |

(duplicate the present table for each outcome)

**Sources used to construct the coding form:**

Gaffney, H., Jolliffe, D., Eggins, E., Ferreira, J. G., Skinner, G., Ariel, B., & Strang, H. (2024). Protocol: The effect of restorative justice interventions for young people on

offending and reoffending: A systematic review. Campbell systematic reviews, 20(2), e1403. https://doi.org/10.1002/cl2.1403

Kethineni, S., Frazier-Kouassi, S., Shigemoto, Y., Jennings, W., Cardwell, S. M., Piquero, A. R., Gay, K., & Sundaravadivelu, D. (2021). PROTOCOL: Effectiveness of parent-engagement programs to reduce truancy and juvenile delinquency: A systematic review. Campbell systematic reviews, 17(3), e1189. https://doi.org/10.1002/cl2.1189
